# Use of bacteriophages for biocontrol of pathogens in food and food-contact surfaces: a systematic review of the literature

**DOI:** 10.1093/sumbio/qvaf005

**Published:** 2025-04-29

**Authors:** Marie N Jayamanne, Antonio C G Foddai

**Affiliations:** Department of Food Science, School of Health and Life Sciences, Teesside University, Middlesbrough, Tees Valley, TS1 3BX, United Kingdom; Department of Food Science, School of Health and Life Sciences, Teesside University, Middlesbrough, Tees Valley, TS1 3BX, United Kingdom

**Keywords:** bacteriophages, foodborne pathogens, biofilms, bio-sanitization, food and food-contact surfaces

## Abstract

Pathogens adhering to food and food-contact surfaces cause high number of foodborne outbreaks worldwide. Their persistent nature and resistance to conventional sanitation methods pose a threat to the food industry and raise food safety issues. Bacteriophages have increasingly emerged as a possible green technology offering host-specific, and natural ability to target and eliminate pathogens from food and food production settings. The aim of this systematic review was to collate the available scientific information on (i) the efficacy of bacteriophages against pathogens in food and food contact-surfaces, and (ii) their stability under diverse processing conditions. An extensive literature search was carried out using three electronic databases: Scopus, Web of Science, and Medline. Forty-nine studies focusing on the reduction of pathogenic bacteria across various food and food-contact surfaces were finally included in this review. Despite some differing results and diverse experimental conditions applied, the overall analysis of the literature confirmed the efficacy of bacteriophages as biocontrol agents in a broad range of food and food surfaces. The findings showed evidence of bacterial reduction against different target pathogens as well as stability under different environmental conditions, including pH and temperature fluctuations normally encountered in food processing environments.

**Sustainability Statement**

Antimicrobial resistance and resistance to chemical disinfectants pose new challenges in modern food production. In recent years, there have been considerable efforts by researchers to identify alternative technologies to be used as more sustainable strategies compared to current industry techniques to ensure food safety and food security. This systematic review gives an insight into the use of bacteriophages to be considered as natural bio-sanitizing agents for the development of new hygienic practices at different food production stages. The topic aims to give substantial contribution to UN Sustainable Development goal 1 (Food security UN SDG2: Zero Hunger) and UN Sustainable Development goal 2 (One Health UN SDG3: Good health and wellbeing).

## Introduction

Food security and food safety represent two key challenges to face the continuously growing global population (O’Sullivan 2023). Microbial contamination represents one of the most common food safety issues reported in many developed and developing countries (Gizaw 2019). Food contaminated with pathogens can cause foodborne illness which has deleterious effects on food security and leads to several socio-economic problems (Noah 2022). The World Health Organization estimates that almost 600 million people get infected and around 420 000 lives are lost due to foodborne illnesses where 40% of these fatalities occur among children under the age of 5. A staggering cost of USD 110 billion is estimated to be lost in productivity and healthcare services each year (WHO 2022). A group of pathogens identified as ‘the big 6’ is responsible for most of the outbreaks worldwide. This includes Norovirus, *Salmonella typhi* (typhoid-like fever), Shiga toxin-producing *Escherichia coli, Shigella* spp., Hepatitis A virus, and *Salmonella* spp. (non-typhoidal) (FDA 2022). Similarly, in Europe, the European Food Safety Authority (EFSA) estimates over 350 000 human cases of foodborne disease each year. The most common sources of foodborne disease are *Campylobacter* spp., *Salmonella* spp., *Yersinia* spp., pathogenic *E. coli*, and *Listeria monocytogenes*. According to the EFSA, the real number of cases is likely to be higher than official records due to underreporting (EFSA 2023). Additionally, pathogenic *Vibrio* spp. have also been increasingly reported as a source of foodborne outbreaks linked to consumption of contaminated sea-food (EFSA 2024).

Because of the potential health risks and associated public health problems, biofilms represent a big threat for the food industry (Shi and Zhu 2009, Abee et al. 2011, Liu et al. 2023). Biofilms are complex and protected communities of microorganisms which are able to grow on surfaces surrounded by self-produced extracellular polymeric substances (EPS) containing a mixture of polysaccharides, proteins, and extracellular DNA. EPS produced by the bacteria are needed to assist the entrapment of nutrients for their survival and make embedded bacteria more resistant than planktonic cells to antibiotics, disinfectants, and sanitizing agents (Folsom and Frank 2006; Abee et al. 2011). It has been estimated that pathogenic biofilms are responsible for nearly 60% of foodborne illnesses and outbreaks (Khan et al. 2023, Liu et al. 2023), and more than one study demonstrates high transmission rates of these bacterial communities to food (Wang et al. 2015, Pang and Hyun-Gyun 2019, Yassoralipour et al. 2023). The ability to form biofilms is well documented in several common foodborne pathogens causing foodborne illness, including *L. monocytogenes, S. aureus, Salmonella* spp., and pathogenic *E. coli* (Bai et al. 2021). Biofilms allow bacteria to bind and persist on a wide range of food processing environments, including ‘abiotic’ and ‘biotic’ surfaces such as wood, polypropylene (PP), glass, plastic, rubber, SS, and food (Carrascosa et al. 2021).

One of the most common strategies adopted in the food industry to minimize the risk of bacterial biofilms is to prevent their formation. This involves a set of aseptic processing procedures, including the regular cleaning, disinfection, and sterilization of equipment and food-contact surfaces before and after use. This is believed to prevent bacterial cells to firmly attach to food surfaces minimizing the risk of bacterial adhesion to key- and high-risk locations. As recently reviewed by Liu et al. (2023), sanitary disinfection programmes regularly applied in the food industry can involve a wide variety of disinfectants including quaternary ammonium compounds, hypochlorite, peroxides such as peracetic acid and hydrogen peroxide, chloramines, iodine, ozone, aldehydes such as formaldehyde, glutaraldehyde, paraformaldehyde, and phenols. However, concerns have been raised about the extensive use of disinfectants due to their potential risk to select resistant strains (Randall et al. 2007, Kim et al. 2018). Heat treatment is another option for the food industry to be used as a measure to decrease the number of harmful bacteria and biofilm populations in food production settings (Park and Yoon 2019, Kim et al. 2020). Steam is probably one of the most promising heat treatment technologies for biofilm inactivation as it demonstrated high efficacy against different foodborne pathogens (Ban et al. 2014) with evidence of high inactivation rates in food (Chang et al. 2010, Park and Yoon 2019) and nonbiological surfaces (Kim et al. 2019).

Due to the high cost and laborious application of conventional sanitation procedures, more sustainable and environmentally friendly control strategies are currently being considered to eliminate and control the formation of harmful biofilms. Rapid raise of antimicrobial resistance (AMR) and increasing evidence of bacterial tolerance to disinfectants also raised questions about the impact of existing biocontrol measurers and has recently accelerated the search for greener and safer strategies (WHO 2024). In this regard, the use of bacteriophages (or phages) as an alternative to antibiotics and disinfectants increasingly emerged over the last two decades (Kutateladze and Adamia 2010, Fernebro 2011, Salmond and Fineran 2015, Moye et al. 2018). Bacteriophages are bacterial-specific viruses able to infect and kill bacteria with the advantage of attacking only bacteria without adverse effect to people (Meaden and Koskella 2013, Kasman and Porter 2024). Bacteriophages are colourless and tasteless living particles that don’t affect sensory properties of food (Aprea et al. 2018, Moye et al. 2018) and their harmless nature and high host-specificality towards target bacteria, make them ideal candidates as a green solution for biocontrol of pathogens (Cristobal-Cueto et al. 2021). Use of bacteriophages including the whole phage body and their bacteriophage-derived bacteriocins as alternative antimicrobial weapons has increasingly been investigated in recent years and still represents an ongoing body of research for many research groups (Gray et al. 2018). Aim of the present systematic review was to collate the most recent scientific literature on the application of bacteriophages against pathogenic biofilms in food and food-contact surfaces and to provide a comprehensive overview of the current evidence on the topic, including benefits and limitations.

## Materials and methods

Literature search. An extensive literature review was conducted in March 2024 using three online electronic databases including Web of Science, Scopus, and Medline available through the Teesside University Library. (Journal Articles & Databases—Food Science and Nutrition—LibGuides at Teesside University). The search was limited to the most recent research articles published in English during the last decade over the period 2010–2024. In line with different objectives of the studies, three independent search strategies were undertaken. The structured vocabulary used was: (i) ‘*efficacy*’ AND ‘*bacteriophages*’ AND ‘*biofilms*’ to find studies on efficacy of bacteriophages; (ii) ‘*environmental*’ AND ‘*persistence*’ OR ‘*stability*’ AND ‘*bacteriophage*s’ were used to find studies on environmental persistence of bacteriophages; and (iii) ‘*pathogen*’ AND ‘*resistance*’ OR ‘*re-growth*’ AND ‘*bacteriophages*’ to find studies on development of resistance in pathogens against bacteriophages.

Eligibility criteria and exclusion criteria. The articles for analysis were selected based on the title, abstract, and keywords of the journal articles. The eligibility criteria to select articles for this systematic review were: original studies published between 2010 and 2014 investigating the use of bacteriophages for sanitization of food and food production environments; studies that presented quantitative levels of efficiency including reduction in viable bacterial counts; studies providing information about the stability of the phage performance under different conditions (i.e. temperature fluctuations, pH levels, chemicals present, and under irradiation); and studies assessing the rate of resistance or re-growth of pathogens after phage treatment. Reviews, book chapters, and studies investigating phage therapy were excluded from the analysis. Studies on bio-sanitization conducted on microorganisms other than foodborne pathogens or studies conducted on bio-sanitization or clinical environments were also excluded.

**Figure 1.**
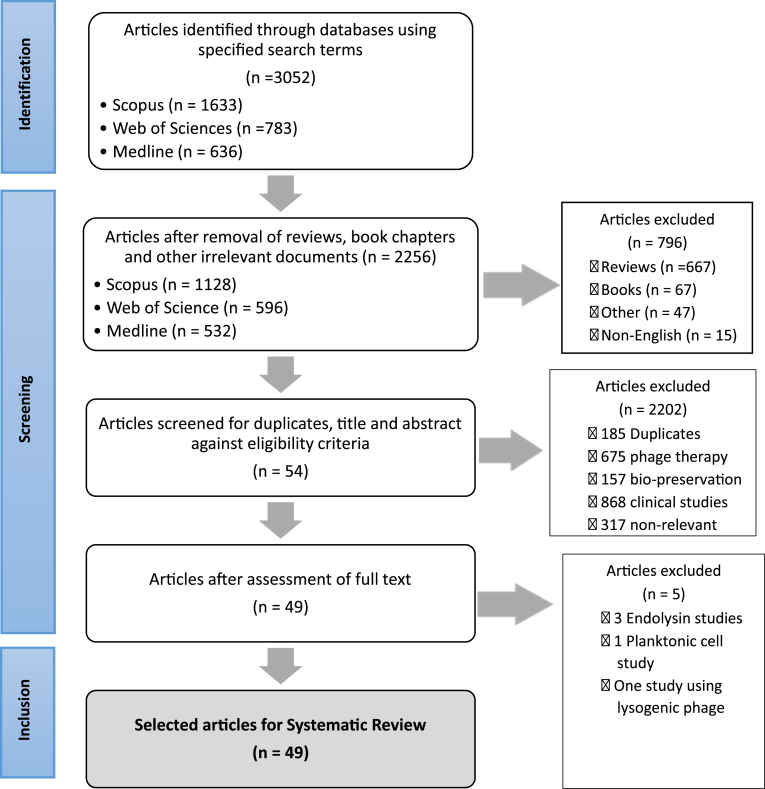
Flowchart of the study selection process showing identification, screening, and inclusion of articles finally retrieved for the systematic review.

Data extraction. In order to minimize bias, data extraction from papers included in this review was carried out independently by two reviewers (M.N.J. and A.C.G.F.). Once preliminary results matching the search terms were obtained, extraction was carried out in three following steps: review articles, book chapters, other documents and articles published in languages other than English were initially identified and removed; research articles were then screened for duplicates which were removed manually; and remaining articles were then assessed against eligibility criteria based on the title and abstract; once screened potential articles were obtained, the full text was assessed for suitability upon the eligibility criteria and included as appropriate to provide an overview of the topic. All these steps were performed following the bibliographic search protocols reported by Page et al. (2021). A manual review of research outputs finally included in this review was performed in parallel by the two reviewers. A visual layout of the data extraction process is represented in Fig. 1.

## Results

Of the 3052 research outputs originally matching the search terms, 54 full journal articles were preliminarily screened for eligibility after duplicates were removed. Subsequent analysis of the full text permitted a final selection of the 49 articles included in this review (Fig. 1) of which 49 studies focused on the efficacy of bacteriophages against pathogens adhering to food surfaces (*n* = 37) and/or food-contact surfaces (*n* = 14); 19 out of 50 studies provided additional information about stability of bacteriophages in different food processing conditions, including pH, temperature, and stability in different food matrices; 8 of the 50 selected studies provided evidence of incomplete inactivation and re-growth of pathogens after phage treatment. The selected literature provided scientific information about efficacy of bacteriophages against different biofilm-forming pathogens including *Salmonella* spp. (*n* = 14), pathogenic *E. coli* (*n* = 11), *L. monocytogenes* (*n* = 15), *Staphylococcus* spp. (*n* = 3), *Campylobacter* spp. (*n* = 4) and *V. parahaemolyticus* (*n* = 3). A summary of the main findings, including the different bacteriophages, their target pathogen, and corresponding inactivation rates from all the studies retrieved is provided in Tables 1 and 2.

**Table 1.** Summary of findings from studies assessing the efficacy of bacteriophages against pathogenic biofilms in food contact materials.

Host	Bacteriophage	Titre (PFU/ml)	MOI	Treatment	Surface	Reduction log_10_ CFU/cm2	Reference
*Salmonella* spp.	LPSTLL, LPST94, and LPST153	10^8^ PFU 10^7^ PFU	N/A	24H at 30°C	Stainless steel (SS)	5.23–6.42 log_10_	Islam et al. (2019)
*Salmonella enterica* serovars Enteritidis and Typhymurium	BP1370, BP1369 (isolated from sewage water)	10^8^ PFU	N/A	2H at 30°C 2H at 10°C	Stainless steel (SS),	2.7 log_10_ at 30°C 3.2 log_10_ at 10°C	Sadekuzzaman et al. (2018)
					Rubber	2.4 log_10_ at 30°C 3.log_10_ at 10°C	
*Listeria monocytogenes*	Phage cocktail (LMPC01, LMPC02, LMPC03			3H at 4°C 6H at 4°C	Polyethylene (PE)	1.04 log_10_ after 3 h 1.59 log_10_ after 6 h	Byun et al. (2024)
					Polypropylene (PP)	1.06 log_10_ after 3 h 1.69 log_10_ after 6 h	
					Stainless steel (SS)	1.16 log_10_ after 3 h 1.76 log_10_ after 6 h	
*Listeria monocytogenes*	Listex™P100	10^9^ PFU	10^3^	4H at 12°C 4H at 32°C	Stainless steel (SS)	2.7–4.9 log_10_	Gutiérrez et al. (2017)
	ListShield™	10^7^ PFU	10			0.31–1.6 log_10_	
*Listeria monocytogenes*	ListSheild™	10^8^ PFU	10^2^	2H at 30°C 2H at 10°C	Stainless steel (SS)	1.9–2.4 log_10_	Sadekuzzaman et al. (2017a)
					Rubber	1 log_10_	
*Listeria monocytogenes*	Listex™P100	10^8^ PFU	N/A	24H at 22°C	Stainless steel wafer (SSW)	Complete inactivation	Iacumin et al. (2016)
*Listeria monocytogenes*	LiMN4L, LiMN4p and LiMN17, individually and mixed	10^9^ PFU	10	1H at 15°C	Clean stainless steel coupon (SSC)	Individual phages: 3–4.5 log_10_ after 75 min Phage mix: 4.6–5.4 log_10_ after 75 min	Arachchi et al. (2013)
					Fish broth layer on stainless steel coupon (FBSSC)	Individual phages: 3–4.5 log_10_ after 75 min Phage mix: 3.8–4.5 log_10_ after 75 min	
		5.8, 6.5 and 7.5 × 10_10_ PFU	N/A		7-day-old biofilms on stainless steel surfaces	Individual phages: 2–3 log_10_ after 1H Phage mix: 2.4 log_10_ after 1H	
		10^9^ PFU	10	1H at 15°C	7-day-old biofilm cells in stainless steel fibres	Individual phages LM 19C09: >5 log_10_ LM 19D03: >5 log_10_ LM 19E03: 4.5 log_10_	
*Listeria monocytogenes*	Listex™P100	10^8^ PFU 10^7^ PFU 10^6^ PFU 10^5^ PFU	10^3^ 10^2^ 10 1	2H, 8H, 24H and 48H at 22°C–24°C	Stainless steel coupon (SSC)	MOI 1000 and 100: ∼3.5 log_10_ after 8H Complete inactivation after 24H MOI 10: Complete inactivation after 24H MOI 1: No inactivation observed	Montañez-Izquierdo et al. (2012)
*Listeria monocytogenes*	Listex™P100	10^9^ PFU	10 10^3^	24H at 22°C	Stainless steel coupon (SSC)	1-day-old biofilm: 5.4 log_10_ 7-day-old biofilm: 3.5 log_10_	Soni and Nannapanemi (2010a)
*Escherichia coli* (O157, O26, O45, O103, O111, O121, O145)	Bacteriophages, used individually and as 21-phage cocktail (P1, P2, P3, P4, P5, P6, P7, P8, P9, P10, P11, P12, P13, P14, P15, P16, P17, P19, P20, P21, J1, J3, J4, J6, J7, J9, J12, J13, J15, J21, J24, J25, J26, J27, J28, J29, J30)	10^9^ PFU	1	16H at 37°C	Stainless steel (SS)	1.9–4.1 log_10_ after 16 h (individual) 3.1–3.7 log_10_ after 16 h (21-phage cocktail)	Jaroni et al. (2023)
					High-density polyethylene (HDPE)	2.3–5.6 log_10_ after 16 h (individual bacteriophages) 2.3–5.3 log_10_ after 16 h (21-phage cocktail)	
*Escherichia coli* MGA-EC-21, MGA-EC-25 and MGA-EC-27	vB_EcoP_PL-01, vB_EcoP_GB-02, vB_EcoP_GB-03 (isolated from poultry liver and ground beef)	10^9^ PFU 10^8^ PFU	10 1	1H at 22°C	Stainless steel (SS)	MOI 10: 2.39–6.79 log_10_ MOI 1: 0.95–2.86 log_10_	González-Gómez et al. (2021)
*Escherichia coli* O145	AZO145A (isolated from cattle feces)	10^10^ PFU	10^3^	24H at 24°C 48H at 24°C 72H at 24°C	Stainless steel coupons	2.9 log_10_ after 24 h 1.9 log_10_ after 48 h 1.9 log_10_ after 72 h	Wang et al. (2020)
*Escherichia coli* O157:H7	BPECO 19 (isolated from sewage sample)	10^8^ PFU	10^2^	2H at 30°C 2H at 10°C	Stainless steel (SS)	3.4–3.8 log_10_ at 30°C 3.0–3.6 log_10_ at 10°C	Sadekuzzaman et al. (2017b)
					Rubber	2.4–2.6 log_10_ at 30°C 2.8–3.0 log_10_ at 10°C	
*Campylobacter jejuni* NCTC 11168 and PT14	CP8, CP30 (isolated from poultry excreta)	10^6^ PFU 10^9^ PFU	10 10^3^	1H, 2H, 4H, 7H and 24H at 37°C	Glass	1–3 log_10_ after 24 h	Siringan et al. (2011)

N/A, not available.

MOI, multiplicity of infection.

**Table 2.** Summary of results from studies focusing on biocontrol of pathogens in food.

Host	Bacteriophage	Titre (PFU/ml)	MOI	Treatment	Food	Reduction log10 CFU/cm2	Reference
**RAW RED MEAT**							
*Staphylococcus aureus*	mSA4 (isolated from retail seafood market)	10^7^ PFU	100	2, 4, 6, and 24H at 4°C	Beef	2.8 log_10_ after 24H	Lambuk et al. (2024)
*Salmonella* Enteritidis, *Salmonella* Typhimurium, and *Escherichia coli* O157:H7	Polyvalent phage PS5	10^8^ PFU	10^4^ 10^3^	2, 4, 6, 24H at 4°C and 24°C	Beef	∼2.5 log_10_ after 6H at 4°C ∼3.5 log_10_ after 6H at 24°C	Duc et al. (2020)
*Campylobacter jejuni*	CJ01 (isolated from retail chicken meat)	10^6^ PFU	10	48H at 4°C	Mutton	1.41 log_10_ after 24H 1.7 log_10_ after 24H	Thung et al. (2019)
*Salmonella enterica* serovar Enteritidis	SE07, (isolated from retail chicken meat)	10^12^ PFU	10^8^	Beef	12H at 4°C 24H at 4°C 48H at 4°C	1.91 log_10_ after 12H 2.06 log_10_ after 24H 2.12 log_10_ after 48H	Thung et al. (2017)
Cocktail of *S almonella enterica, S almonella* Heidelberg, *almonella* Newport, and *Salmonella Enteritidis*	Salmonelex™ (mix of phages S16 and Felix-O1a)	10^8^ PFU 10^7^ PFU	10 1	6H at 4°C 18H at 4°C	Ground beef Ground pork	Ground beef: MOI 10: 1 log_10_ MOI 1: 0.7 log_10_ Ground pork: MOI 10 and 1: 0.8 log_10_	Yeh et al. (2017)
*Escherichia coli* O157	T1, T4, T5, O1 (used alone and as a mix)	10^8^ PFU	10^3^	144 H at 4°C 6H at 22°C	Beef	T5: ∼2.5 log_10_ after 144H T1: ∼1.5 log_10_ after 144H T4: ∼1.5 log_10_ after 144H O1: ∼1 log_10_ after 144H Mix: ∼1.5 log_10_ after 144H T5: ∼3 log_10_ after 6H T1: ∼2 log_10_ after 6H T4: ∼2 log_10_ after 6H O1: ∼1 log_10_ after 6H Mix: ∼1.5 log_10_ after 6H	Liu et al. (2015)
				3H at 37°C		T5: ∼1.5 log_10_ after 1.5H T1: ∼1 log_10_ after 3H T4: ∼1.5 log_10_ after 3H O1: ∼1 log_10_ after 1.5H Mix: ∼1 log_10_ after 3H	
		10^6^ PFU	10	144 H at 4°C 6H at 22°C 3H at 37°C		T5: <1 log_10_ after 144H T1: <1 log_10_ after 144H T4: <1 log_10_ after 144H O1: <1 log_10_ after 144H Mix: <1 log_10_ after 144H T5: ∼1 log_10_ after 6H T1: ∼1 log_10_ after 6H T4: ∼1 log_10_ after 6H O1: ∼1 log_10_ after 6H Mix: ∼1 log_10_ after 6H T5: <1 log_10_ after 2H T1: ∼1 log_10_ after 2H T4: ∼1 log_10_ after 2H O1: ∼1 log_10_ after 2H Mix: ∼1 log_10_ after 2H	
**RAW POULTRY and EGGS**							
*Salmonella* Enteritidis, *Salmonella* Typhimurium,	Polyvalent phage PS5	10^8^ PFU	10^4^ 10^3^	2, 4, 6, 24H at 4°C 2, 4, 6, 24H at 24°C	Chicken skin	1.2 log_10_ after 2H at 4°C 2.4 log_10_ after 2H at 24°C	Duc et al. (2020)
*Campylobacter jejuni*	F356 and F357 (isolated from free-range poultry farms) individually and mix	10^7^ PFU	10^3^	24H at 5°C	Chicken skin	F356 alone: 0.49 log_10_ F357 alone: 0.55 log_10_ Mix: 0.73 log_10_	Zampara et al. (2017)
*Salmonella* Typhimurium, *Salmonella* Heidelberg, and *Salmonella* Enteritidis	SalmoFresh™ used	10^9^ PFU	10^6^	24H at 4°C	Chicken skin	1 log_10_	Sukumaran et al. (2015)
*Salmonella* Enteritidis spp.	phiSE 7 (isolated from a chicken farm)	10^9^ PFU	10^4^ 10	30 min at RT 16H at 6°C	Chicken skin	1 log_10_ 1 log_10_	Hungaro et al. (2013)
*Salmonella enterica* serovars	SEPL01, SEPL13, SEPL20 (isolated from a sewage—mix)	10^11^ PFU 10^9^ PFU	10^4^ 10^2^	72H at 4°C	Chicken breast	MO1 10^4^ 2.72 log_10_ after 3 days MO1 10^2^ 1.47 log_10_ after 3 days	Kokab et al. (2022)
*Salmonella* spp.	LPSTLL, LPST94, and LPST153 (mix)	10^7^ PFU 10^6^ PFU	10^4^ 10^3^	1, 3, 6, 12, 25H at 25°C	Chicken breast	MOI: 10^4^ Complete inactivation after 3H MOI: 10^3^ Complete inactivation after 6H	Islam et al. (2019)
				1, 3, 6, 12, 25H at 4°C		MOI: 10^4^ Complete inactivation after 3H MOI: 10^3^ Complete inactivation after 3H	
*Salmonella* Enteritidis	PA13076 and PC2184 (isolated from chicken sewage—mix)	10^9^ PFU	10^4^	1–5H at 4°C 1–5H at 25°C	Chicken breast	1.65 log_10_ after 1H at 4°C and 25°C	Bao et al. (2015)
*Salmonella* Typhimurium ATCC14028 (UA1872) and *Salmonella* Enteritidis LK5 (UA1894)	UAB_Phi20, UAB_Phi78, and UAB_Phi87–mix	10^9^ PFU	10^3^	7 days at 4°C	Chicken breast	UA1872: 2.2 log_10_ after 7H UA1894: 1.4 log_10_ after 5H	Spricigo et al. (2013)
Coagulase-negative *Staphylococcus* spp.	CoNShP-3 (isolated from a sewage)	10^8^ PFU	10^2^	1, 2, 5, 7 days at 4°C 3, 7, 14, 21 and 31 days at -20°C	Chicken breast cuts	Treatment at 4°C: 5.18 log_10_ after 7 days Treatment at -20°C: 0.46 log_10_ after 3 days 1.31 log_10_ after 30 days	Sofy et al. (2020)
*Salmonella* Typhimurium, *Salmonella* Heidelberg, and *Salmonella* Enteritidis	SalmoFresh™ used alone	10^9^ PFU 10^8^ PFU	10^6^ 10^5^	1 and 7 days at 4°C	Chicken breast	PA: 1.1 log_10_ after 1 day PA: 1 log_10_ after 7 days PA: 1.1 log_10_ after 1 day PA: 1 log_10_ after 7 days	Sukumaran et al. (2015)
*Listeria monocytogenes*	Commercial ListSheild™	10^8^ PFU	10^3^	1H at 4°C 24H at 4°C 48H at 4°C 72H at 4°C	Chicken breast	0.56 log_10_ after 1H 0.84 log_10_ after 24H 0.46 log_10_ after 48H 0.10 log_10_ after 72H	Yang et al. (2017)
*Campylobacter jejuni*	CJ01 (isolated from retail chicken meat)	10^6^ PFU	10	48H at 4°C	Diced chicken meat	1.37 log_10_ after 24H 1.68 log_10_ after 48H	Thung et al. (2019)
*Salmonella enterica* serovar Enteritidis	SE07, (isolated from retail chicken meat)	10^11^ PFU	10^5^	12H at 4°C 24H at 4°C 48H at 4°C	Diced chicken meat	1.82 log_10_ 1.95 log_10_ 2.02 log_10_	Thung et al. (2017)
Cocktail of *Salmonella enterica, Salmonella* Heidelberg, *Salmonella* Newport, and *Salmonella Enteritidis*	Salmonelex™ (mix of phages S16 and Felix-O1a)	10^8^ PFU 10^7^ PFU	10 1	30 min at 4°C 6H at 4°C	Ground chicken Ground turkey	Ground chicken: MOI 1; 30 min: no significant reduction MOI 10; 30 min: 0.8 log_10_ MOI 10; 6H: 1.1 log_10_	Yeh et al. (2017)
						Ground turkey: MOI 10: 0.9 log_10_ MOI 1: 0.7 log_10_	
*Salmonella* serovar Thompson	CAU-STP-1, CAU-STP-2 (isolated from river water—mix)	10^10^ PFU 10^8^ PFU 10^7^ PFU	10^5^ 10^4^ 10^3^	7 days at 20°C 7 days at 10°C	Eggshell	MOI 10^5^: 0.33 log_10_ after 7 days at 20°C 2.5 log_10_ 7 days at 10°C MOI 10^4^: 0.24 log_10_ after 7 days at 20°C 0.96 log_10_ 7 days at 10°C MOI 10^3^: 0.10 log_10_ after 7 days at 20°C 0.26 log_10_ 7 days at 10°C	Ashrafudoulla et al. (2023)
*Salmonella enterica* serovar Enteritidis	SE07, (isolated from retail chicken meat)	10^12^ PFU	10^7^	12H at 4°C 24H at 4°C 48H at 4°C	Eggshell	1.7 log_10_ after 12H 1.84 log_10_ after 24H 1.89 log_10_ after 48H	Thung et al. (2017)
*Salmonella* Typhimurium ATCC14028 (UA1872) and S*almonella* Enteritidis LK5 (UA1894)	UAB_Phi20, UAB_Phi78, and UAB_Phi87–mix	10^10^ PFU	10^3^	3H at RT	Eggshell—surviving bacterial detected on broken eggs	UA1872: 0.9 log_10_ UA1894: 0.9 log_10_	Spricigo et al. (2013)
*Mix of Salmonella* Enteritidis, *Salmonella* Typhimurium, and *Escherichia coli* O157:H7	Polyvalent phage PS5	10^10^ PFU	10^4^	2, 4, 6, and 24H at 4°C and 24°C	Liquid eggs	∼1 log_10_ after 2H at 4°C ∼2 log_10_ after 2H at 24°C	Duc et al. (2020)
*Salmonella* Typhimurium	FO1-E2	10^8^ PFU	10^4^	1, 2, 5, and 6 days at 15°C	Egg yolk	No significant reduction	Guenther et al. (2012)
**RAW SEAFOOD**							
*Vibrio parahaemolyticus*	vB_VpaS_OMN (isolated from atlantic Sea water)	10^6^ PFU	10^2^	1H, 6H, 12H, 24H, 48H, and 72H at 15°C	Oysters	0.83 log_10_ after 6H 2.9 log_10_ after 48H 99% inactivation after 72H	Zhang et al. (2018)
*Vibrio parahaemolyticus*	pVp-1	10^7^ PFU	10	0.5, 1, 2, 3, 6, 12, 24, 36, 48, and 72H at RT with ice cubes	Oysters	∼2 log_10_ after 2H ∼2.5 log_10_ after 6H and 12H ∼3 log_10_ after 24H ∼4.5 log_10_ after 36H ∼6 log_10_ after 48H and 72H	Jun et al. (2014)
*Salmonella* Typhimurium	FO1-E2	10^8^ PFU	10^4^	1, 2, 5, and 6 days at 8°C	Mixed seafood	Complete inactivation after 24H	Guenther et al. (2012)
				1, 2, 5, 6 days at 15°C	Mixed seafood	1 log_10_ after 2 days	
*Listeria monocytogenes*	Listex™P100	10^9^ PFU 10^8^ PFU 10^7^ PFU 10^9^ PFU 10^9^ PFU	10^5^ 10^4^ 10^3^ 10^5^ 10^5^	2H at 22°C 15, 30, 60, and 120 min at 22°C 1–10 days at 4°C and 10°C	Catfish fillet (5 g)	Treatment at 22°C for 2H: MOI 10^5^: 0.8 log_10_ MOI 10^4^: 0.4 log_10_ MOI 10^3^: no reduction Treatment at 22°C: 15 min: 0.8 log_10_ 30 min: 1.3 log_10_ 60 min: 1.6 log_10_ 120 min: 1.6 log_10_ 10-day shelf life at 4°C: 1.5 log_10_ 10-day shelf life at 10°C: 1.7–2 log_10_	Soni et al. (2010)
		10^9^ PFU	10^5^	30 min at 22°C 2H at 22°C	Large catfish fillet (180–220 g)	1.7 log_10_ after 30 min 2.1 log_10_ after 2H	
*Listeria monocytogenes*	Listex™P100	10^10^ PFU 10^9^ PFU 10^8^ PFU 10^7^ PFU 10^6^ PFU	10^6^ 10^5^ 10^4^ 10^3^ 10^2^	2H at 22°C	Salmon fillets	Treatment at 22°C for 2H: MOI 10^6^: 3.5 log_10_ MOI 10^5^: 2 log_10_ MOI 10^4^: 1.2 log_10_ MOI 10^3^: 0.5 log_10_	Soni and Nannapanemi (2010b)
				30 min and 2H at 4°C and 22°C		MOI 1: no significant reduction 4 log 10 CFU/g: 3 log_10_ after 30 min and 120 min at 4 and 22°C 3 log 10 CFU/g: 2.5 log_10_ after 30 min and 120 min at 4°C 2.9 log_10_ after 30 min and 120 min at 22°C 2 log 10 CFU/g: 2.5 log_10_ after 30 min and 120 min at 4°C 2.9 log_10_ after 30 min and 120 min at 22°C	
				1–10 days at 4°C		10-day shelf life at 4°C: Stable for 4 days at 4C 0.6 log_10_ after 10 days	
**READY-TO-EAT FOOD**							
*Vibrio parahaemolyticus*	VPT02 (isolated from market oysters	10^5^ PFU	10 1	6H at 25°C 6H at 5°C	RTE raw fish slices	Treatment at 25°C: MOI 10: 2 log_10_ MOI 1: ∼1.8 log_10_ Treatment at 5°C: MOI 10: 1.5 log_10_ MOI 1: ∼1.3 log_10_	You et al. (2021)
*Listeria monocytogenes*	Listex™P100	10^8^ PFU	10^6^ 10^5^ 10^4^	24H at 20°C 24H at 10°C 24H at 4°C	Dry-cured ham slices	MOI 10^6^ and 10^5^: Complete inactivation at 4°C, 10°C and 20°C MOI 10^4^: 3.7, 1.8 and 1 log_10_ reduction after 2H hours at 4°C, 10°C and 20°C	Iacumin et al. (2016)
		10^8^ PFU 10^7^ PFU 10^6^ PFU 10^5^ PFU 10^4^ PFU	10^6^ 10^5^ 10^4^ 10^3^ 10^2^ 10 1	24H at 4°C		MOI 10^6^ to 10^3^ Complete inactivation MOI 10^2^ to 1 Incomplete inactivation: 0.1, I, 1.5 log_10_ reduction at MOI 100, 10 and 1	
*Salmonella* Typhimurium	FO1-E2	10^8^ PFU	10^4^	1, 2, 5, and 6 days at 15°C	Hotdog Sliced turkey	Complete inactivation after 2 days Complete inactivation after 2 days	Guenther et al. (2012)
*Listeria monocytogenes*	FWLLm1 (isolated from sheep faeces)	10^7^ PFU 10^6^ PFU	10^2^ 10^4^ 10^2^	25 days at 30°C 25 days at 5°C	Sliced chicken breast	2.5 log_10_ after 48H 1.5 log_10_ after 7 days 1.5 log_10_ after 7 days	Bigot et al. (2011)
*Listeria monocytogenes*	Listex™P100	10^9^ PFU	10^4^	30 min at 21°C 7 days at 4°C	Minas Frescal Coalho cheese	2.3 log_10_ after 30 min 21°C 1 log_10_ after 7 days at 4°C 2.1 log_10_ after 30 min at 21°C 0.8 log_10_ after 7 days at 4°C	Silva et al. (2014)
*Listeria monocytogenes*	A511	10^9^ PFU 10^8^ PFU	10^7^ 10^6^ 10^5^	1–22 days at 6°C MOI 10^6^–10^7^: single dose at T0 MO1 10^5^ 1 dose: day 1 2 doses: day 0, 6	White-mold cheese	Low contamination (1–2 log_10_CFU/cm^2^): Complete inactivation after 6 days High contamination (3 log_10_ CFU/cm^2^) 1 dose: 2 log_10_ after 9 days 2 doses: 3 log_10_ after 9 days	Guenther and Loessner (2011)
**FRUIT AND VEGETABLES**							
*Salmonella* serovar Newport	CGG3-1, CGG3-2, CGG3-4.1 and CGG3-4.1 (isolated from sewage water—mix)	10^8^ PFU 10^6^ PFU	10^5^ 10^3^	1H at 25°C	Cherry tomatoes	MO1 10^5^: 3 log_10_ after 1, 2, 3 days 4.4 log_10_ after 4 days MO1 10^3^ 2 log_10_ after 1, 2, 3 and 4 days	El-Dougdoug et al. (2019)
*Staphylococcus aureus*	mSA4 (isolated from retail seafood market)	10^8^ PFU 10^7^ PFU	10^3^ 10^2^	2, 4, 6, and 24H at 4°C	Iceberg lettuce	Iceberg lettuce: MOI 10^3^: 3.2 log_10_ after 24H	Lambuk et al. (2024)
*Salmonella* Enteritidis, *Salmonella* Typhimurium, and *Escherichia coli* O157:H7	Polyvalent phage PS5	10^8^ PFU	10^4^ 10^3^	2, 4, 6, and 24H at 4°C and 24°C	Lettuce	Complete inactivation after 2H at 4°C Complete inactivation after 2H at 24°C	Duc et al. (2020)
*Salmonella enterica* serovars	BP1370, BP1369 (mix—isolated from sewage water)	10^8^ PFU	10^2^	2H at 10°C 2H at 20°C 2H at 25°C	Lettuce	>1 log_10_	Sadekuzzaman et al. (2018)
*Salmonella* Typhimurium ATCC14028 (UA1872) and S. Enteritidis LK5 (UA1894)	UAB_Phi20, UAB_Phi78, and UAB_Phi87–mix	10^9^ PFU	10^4^	0, 30 min and 60 min at RT	Lettuce	UA1872: 3.4 log_10_ after 30 min 3.9 log_10_ after 60 min UA1872: 1.9 log_10_ after 30 min 2.2 log_10_ after 60min	Spricigo et al. (2013)
*Escherichia coli* O157:H7	VE04, VE05, VE07 (individually)	10^8^ PFU	10^2^	2H at 10°C	Roman lettuce	2.6–6 log_10_	Lu et al. (2022)
*Escherichia coli* O157:H7	BPECO 19 (isolated from sewage sample)	10^8^ PFU	10^2^	2H at 10°C 2H at 20°C 2H at 25°C	Lettuce	2.4 log_10_ after treatment at 25°C, 2.2 log_10_ after treatment at 22°C, and 2.6 log_10_ after treatment at 10°C	Sadekuzzaman et al. (2017b)
*Escherichia coli* O157:H7	Commercial EcoShield™	10^9^PFU	10^5^	24H at 4°C	Lettuce	1.1 log_10_	Ferguson et al. (2013)
*Listeria monocytogenes*	Commercial ListSheild™	10^8^ PFU	10^2^	2H at 10°C 2H at 20°C 2H at 25°C	Lettuce	0.7 log_10_	Sadekuzzaman et al. (2017a)^a^
*Listeria monocytogenes*	Phage cocktail (LMPC01, LMPC02, LMPC03)	10^8^ PFU	10^4^	1 to 7days at 4°C	Celery	1.63 log_10_ after 5 days	Byun et al. (2024)
*Escherichia coli* O157:H7,	Commercial EcoShield	10^10^ PFU	10^4^	24H at 10°C	Strawbery Cantaloupe Broccoli	0.53 log_10_ 0.74 log_10_ 0.68 log_10_	Magnone et al. (2013)
*Salmonella spp*,	Commercial The SalmoFresh	10^10^ PFU	10^4^	24H at 10°C	Strawbery Cantaloupe Broccoli	0.53 log_10_ 0.78 log_10_ 2.79 log_10_	
*Shigella* spp	Commercial ShigActive	10^10^ PFU	10^4^	24H at 10°C	Strawbery Cantaloupe Broccoli	0.34 log_10_ 0.75 log_10_ 0.32 log_10_	
*Listeria monocytogenes*	Listex™P100	10^8^ PFU	10^3^	8 days at 10°C	Melon slices Pear slices Apple slices	1.5 log_10_ 0.6 log_10_ No significant reduction	Oliveira et al. (2014)
**LIQUID FOOD AND DRINKS**							
*Staphylococcus aureus*	Vb-SAV-SDQ	10^7^ PFU	10	24H at 37°C 48H at 37°C	Milk	24H exposure: 54% reduction after 24H 71% reduction after 72H 48H exposure 69% reduction after 24H 95% reduction after 48H	Song et al. (2021)
*Salmonella* Enteritidis, *Salmonella* Typhimurium, and *Escherichia coli* O157:H7	Polyvalent phage PS5	10^8^ PFU	10^4^ 10^3^	2, 4, 6, and 24H at 4°C and 24°C	Pasteurized milk	∼2 log_10_ after 2H at 4°C ∼2 log_10_ after 2H at 24°C	Duc et al. (2020)
*Salmonella* Enteritidis	PA13076 and PC2184 (isolated from chicken sewage—mix)	10^9^ PFU	10^4^	5H at 4°C 5H at 25°C	Pasteurized whole milk	Milk: 3.89 log_10_ after 1H at 4°C Complete inactivation after 1H at 25°C	Bao et al. (2015)
*Staphylococcus aureus*	mSA4 (isolated from retail seafood market)	10^8^ PFU 10^7^PFU	10^3^ 10^2^	2, 4, 6, and 24H at 4°C	Chocolate milk,	Chocolate milk: MOI 10^3^: 2.1 log_10_ after 24H MOI 10^2^: 1.9 log_10_ after 24H	Lambuk et al. (2024)
*Salmonella* Typhimurium	FO1-E2	10^8^ PFU	10^4^	1, 2, 5, and 6 days at 8°C	Chocolate milk	Complete inactivation after 24H	Guenther et al. (2012)
				1, 2, 5, and 6 days at 15°C	Chocolate milk	Complete inactivation after 2 days	
Salmonella enterica serovar Enteritidis	SE07, (isolated from retail chicken meat)	10^11^ PFU	10^5^	12H at 4°C 24H at 4°C 48H at 4°C	Fruit juice	2 log_10_ after 12H 2.14 log_10_ after 24H 2.2 log_10_ after 48H	Thung et al. (2017)
*Listeria monocytogenes*	Listex™P100	10^8^ PFU	1000	8 days at 10°C	Melon juice, Pear juice, and Apple juice	0.6 log_10_ 3 log_10_ No significant reduction	Oliveira et al. (2014)

MOI, multiplicity of infection.

### Inhibition of pathogenic biofilm in food contact materials

Fourteen selected studies attempted to assess the efficacy of bacteriophages against biofilm forming pathogens on food contact materials. Materials tested were SS, including either cleaned (SSC) and fish broth layer (FBSSC), rubber, PE, PP, HDPE, and glass. Key findings of these studies are reported below in reverse chronological order as presented in Table 1.

Islam et al. (2019) and Sadekuzzaman et al. (2018) conducted studies on the efficiency of different phages against *Salmonella* spp. Islam et al. (2019) assessed the biofilm removal ability of a phage cocktail containing three phages (LPSTLL, LPST94, and LPST153) against diverse *Salmonella* serovars grown individually and as a mix on SS coupons; the overall log_10_ CFU reduction ranged 5.23–6.42. Sadekuzzaman et al. (2018) used two bacteriophages BP1370 and BP1369 against biofilms of *Salmonella enterica* serovars Enteritidis and Typhimurium grown on SS and rubber; the phage treatment applied at a concentration of 8 log_10_ PFU/ml for 2H at 30°C and 10°C achieved 2.7 and 3.2 log_10_ CFU/cm^2^ viable count reduction on SS surface, and 2.4 and 3 log_10_ CFU/cm^2^ on rubber surfaces, respectively.

Seven studies, including Montañez-Izquierdo et al. (2012), Arachchi et al. (2013), Gutiérrez et al. (2017), Iacumin et al. (2016), Sadekuzzaman et al. (2017a), Byun et al. (2024), and Soni and Nannapeni (2010a), evaluated the use of bacteriophages for eradicating *L. monocytogenes* biofilms on various food contact materials. Byun et al. (2024) tested a phage cocktail prepared combining LMPC01, LMPC02, and LMPC03 phages against listeria-biofilms prepared on PE, PP, and SS; longer phage treatment applied for 6H proved to be more successful than that applied for 3H; bacterial reduction observed for phage treatments applied at 4°C for 3H and 6H ranged 1.04–1.16 Log_10_ CFU/cm^2^ and 1.59–1.76 log_10_ CFU/cm^2^, respectively. Gutiérrez et al. (2017) tested the antimicrobial efficiency of two commercial listeriophage products ListShield™ and Listex™ P100 applied to SS surfaces treated 4 h at 12°C and 32°C; the study reported higher level of reduction in viable cell counts obtained using Listex™ P100 compared to ListShield™ applied alongside; Listex™ P100 was able to reduce the viable cell counts by 2.7–4.9 log_10_ CFU/cm^2^; ListShield™ achieved reduction rates ranging between 0.31 and 1.6 log_10_ CFU/cm^2^. Conversely, higher bacterial reduction achieved by the commercial listeriophage product ListShield™ was reported by Sadekuzzaman et al. (2017a); the product applied to SS and rubber for 2 h at 10°C and 30°C achieved bacterial reductions of 1.9–2.4 log_10_ CFU/cm^2^ on the SS and ∼1.0 log_10_ CFU/cm^2^ on rubber. Iacumin et al. (2016) assessed the biofilm removal activity of the commercial listeriophage-based product Listex™ P100 applied to SSW and reported complete inactivation after phage treatment applied at 20°C for 24H. Arachchi et al. (2013) investigated the antimicrobial activity of three bacteriophages (LiMN4L, LiMN4p, and LiMN17) used alone or as a cocktail against 3 seafood-borne *L. monocytogenes* strains grown on (i) fish broth layer; (ii) on stainless steel coupon (FBSSC); and (iii) clean SSC for 72H at 30°C and 7 days at 15°C ± 1°C; the phage cocktail initially applied to 72H-old biofilms formed on FBSSC and SSC demonstrated much higher inactivation power (3.8–4.5 and 4.6–5.4 log_10_ CFU/cm^2^, respectively), than individual applications carried out in parallel (3.8–4.5 log_10_ reduction units observed for both FBSS and SSC); 7-day *L. monocytogenes* biofilms proved to be more resistant as expected: three consecutive applications were necessary to achieve substantial decrease (2–3 log_10_ units) of the original 4 log_10_ CFU bacterial load. Montañez-Izquierdo et al. (2012) evaluated the effectiveness of phage P100 for biocontrol of *L. monocytogenes* biofilms allowed to develop for 72H at room temperature on SS surfaces then treated with different concentrations of phage (5, 6, 7, and 8 log_10_ PFU/ml); the level of inactivation was found to be dependent on the phage/bacterial cell ratio (see Table 1); complete inactivation was observed where biofilms were treated at high phage titre (MOI 10^3^ to 10) whereas not significant reduction was observed at lower phage dose. Similarly, Soni and Nannapaneni (2010a) tested the effectiveness of phage P100 in controlling 1-day and 7-day listeria*-*biofilms grown on SS surfaces at 22°C; the phage P100 treatment significantly reduced bacterial populations in both cases and achieved 3.5–5.4 log_10_/cm^2^ reduction, respectively; as summarized in Table 1, bacterial inactivation of 7-day-old and 1 day-old biofilm required different phage titres (MOI 10^3^ and 10, respectively); higher phage concentration was necessary to achieve substantial bacterial reduction in 7-day-old biofilms compared to what applied to 1-day-old biofilms.

Wang et al. (2020), González-Gómez et al. (2021), Jaroni et al. (2023), and Sadekuzzaman et al. (2017b) evaluated the efficacy of bacteriophage-based treatments against *E. coli* biofilms. Jaroni et al. (2023), evaluated the biofilm removal of a large number of bacteriophage isolates (P1, P2, P3, P4, P5, P6, P7, P8, P9, P10, P11, P12, P13, P14, P15, P16, P17, P19, P20, P21, J1, J3, J4, J6, J7, J9, J12, J13, J15, J21, J24, J25, J26, J27, J28, J29, and J30) used individually and as cocktails against different STEC strains (O157, O26, O45, O103, O111, O121, and O145) grown on SS and HDPE coupons; results showed a significant reduction in viable counts in both cases; average biofilm reduction ranged 1.9–4.1 log_10_ CFU/cm^2^ on SS surfaces, and 2.3–5.6 log_10_ CFU/cm^2^ on HDPE surfaces; results didn’t show any statistical difference between individual-phage and phage cocktail applications. González-Gómez et al. (2021) tested the antibiofilm activity of three bacteriophage (vB_EcoP_PL-01, vB_EcoP_GB-02, and vB_EcoP_GB-03), originally isolated from poultry liver and ground beef, against three *E. coli* strains; the results showed a greater statistical difference depending on bacteriophage concentration used for biofilm removal; treatment with 10^9^ PFU/ml phage concentration showed the highest level of bacterial reduction ranging 2.39–6.79 log_10_ CFU/cm^2^; lower reduction rates (0.95–2.86 log_10_ CFU/cm^2^) was observed for biofilms treated with phage concentration of 10^8^ PFU/ml (Table 1); there was also no significant difference between treatments involving individual phages or phage cocktails. Wang et al. (2020) tested the phage AZO145A against biofilms formed by pathogenic bacteria Shiga Toxigenic *Escherichia coli* (STEC) O145: H25 cultivated for 24, 48, and 72H on SS coupons; inactivation rates observed on 24H-old, 48H-old, and 72H-old biofilms were 2.9, 1.9, and 1.9 log_10_ CFU/coupon, respectively. Sadekuzzaman et al. (2017b) assessed the efficacy of bacteriophage BPECO 19 against *Escherichia coli* O157: H7 biofilms prepared on SS, rubber and treated with a phage suspension of 10^8^ PFU/ml for 2H at 30°C and 10°C; after 2H of treatment, *E. coli* O157: H7 populations grown on SS were reduced by $\sim$3.5 log_10_ CFU/cm^2^ at 30°C and by $\sim$3.2 log_10_ CFU/cm^2^ at 10°C; corresponding reductions observed on treated rubber surfaces were $\sim$2.5 log_10_ CFU/cm^2^ for biofilm grown at 30°C and $\sim$2.9 log_10_ CFU/cm^2^ for biofilms grown at 10°C.

Different from other foodborne pathogens, limited information seems to be currently available on the literature about biocontrol of biofilms formed by *C. jejunii*. Siringan et al. (2011) tested the efficacy of bacteriophages bacteriophage CP8 and CP30 against *C. jejuni* NCTC 11168 and PT14 cultivated on glass surfaces under microaerophilic conditions; bacterial reductions observed 24H post-infection ranged 1–3 log_10_ CFU/cm^2.^

### Biocontrol of pathogenic bacteria adhering food

Significant amount of literature published over the last 14 years evaluated the efficacy of bacteriophages for biocontrol of pathogens in food. Forty-seven selected studies attempted to assess the antimicrobial activity of bacteriophage in different categories of food including raw red meat (*n* = 6), poultry (*n* = 14), eggs (*n* = 5), sea-food (*n* = 6), ready-to-eat food (*n* = 6), fruit and vegetables, (*n* = 13), and liquid food and/or drinks (*n* = 7). A summary of the main findings from all the studies included in this section are reported in the same reverse chronological order used in Table 2. Information relating to the different categories of food assessed is summarized below.

### Raw red meat

Thung et al. (2017), Yeh et al. (2017), Thung et al. (2019), Duc et al. (2020), Lambuk et al. (2024), and Liu et al. (2015) documented the phage-based bacterial inactivation raw red meat. Lambuk et al. (2024) reported 2.8 log_10_ CFU reduction of *Staphylococcus aureus* from meat treated with phage mSA4 after 24H at 4°C. Duc et al. (2020) tested the inactivation abilities of a polyvalent phage PS5 applied overtime at 4°C and 24°C against *Sa*. Enteritidis, *Sa*. Typhimurium, and *E. coli* O157: H7 in beef; the study reported significant reductions of viable counts of all the three bacterial hosts with reduction rates of ∼2.5 log_10_ after phage treatment applied for 6H at 4°C, and ∼3.5 log_10_ for treatments applied in parallel for 6H at 24°C. Thung et al. (2019) evaluated bacteriophage CJ01 against *C. jejuni* in mutton and reported reductions of 1.41 log_10_ CFU/g and 1.70 log_10_ CFU/g after 24H and 48H at refrigeration temperature (4°C). Thung et al. (2017) assessed the antimicrobial activity of a new phage (SE07), originally isolated from retail chicken meat, against *Sa. enterica* serovar Enteritidis in beef; the study demonstrated higher inactivation rates when the phage treatment was applied for prolonged periods; reduction of viable counts observed after phage treatment applied for 12H, 24H, and 48H at 4°C were 1.91 log_10_ 2.06 log_10_ CFU and 2.12 log_10_ CFU. Yeh et al. (2017) assessed the antimicrobial efficacy of the commercial phage-based product Salmonelex™ (mix of phages S16 and Felix-O1a) applied at 10^7^ and 10^8^ PFU/ml against a cocktail of *Sa. enterica, Sa. Heidelberg, Sa. Newport*, and *Sa. Enteritidis* in ground pork and beef; higher phage doses achieved higher level of inactivation; bacteriophage application reduced *Salmonella* spp. by 1 and 0.8 log_10_ CFU/g in ground beef and ground pork, respectively. Liu et al. (2015) reported the antimicrobial activities of four bacteriophages (T1, T4, T5, and O1) used alone and as a mix against *E. coli* O157; the study considered different phage/cells ratios (MOI 10^3^ and 10); higher phage dose generally attracted higher level of bacterial inactivation; overall inactivation rates ranged between 1 and 3 log_10_ CFU/g; Phages T1, T4, and T5 were generally more effective than Phage 01 and achieved higher levels of bacterial inactivation when used individually rather as a mix (Table 2)

### Raw poultry and eggs

Twenty selected studies attempted to assess phage-based bacterial removal from raw poultry products. Product tested included, chicken skin (*n* = 4), chicken breast (*n* = 7), diced chicken meat (*n* = 1), ground chicken or turkey meat (*n* = 2), and eggs (*n* = 5). A summary of the main findings is provided in the corresponding section of Table 2.

Duc et al. (2020), Sukumaran et al. (2015), Zampara et al. (2017), and Hungaro et al. (2013) evaluated the phage-based removal of pathogens in chicken skin. Duc et al. (2023) reported significant reduction achieved by the polyvalent phage PS5 against *Sa*. Enteritidis and *Sa*. Typhimurium; inactivation rates observed were 1.2 log_10_ and 2.4 log_10_ after 2H at 4°C and 24°C, respectively. Zampara et al. (2017) tested the bacterial removal of two phages (F356 and F357) used alone and as a mix against *C. jejuni;* the poly-phage treatment applied at 5°C demonstrated statistically higher level of inactivation than single phage application and achieved 0.73 log_10_ CFU reduction of *C. jejuni* compared to 0.49 and 0.55 log_10_ CFU reductions as achieved by F356 and F357 used alone. Sukumaran et al. (2015) evaluated the commercial bacteriophage preparation SalmoFresh™(Intralytix Inc., USA) containing 6 lytic phages against a mix of *Salmonella* spp. strains (*Sa*. Typhimurium, *Sa*. Heidelberg, and *S a*. Enteritidis) and reported 1 log_10_ CFU reduction after 24H at 4°C. Similar rates of inactivation were also reported by Hungaro et al. (2013) who evaluated the phage phiSE 7, originally isolated from a chicken farm, against *Salmonella* Enteritidis in chicken skin; ∼1 log_10_ CFU reduction was observed after phage treatment applied at room temperature and 4°C.

Five studies assessed removal abilities of new bacteriophage isolates tested against *Salmonella* spp. (Spricigo et al. 2013, Bao et al. 2015, Islam et al. 2019, Kokab et al. 2022) and *Staphylococcus* spp. (Sofy et al. 2020) in chicken breast. Kokab et al. (2022) tested a mix of three phage isolates (SEPL01, SEPL13, and SEPL20) against different *Sa*. Enterica serovars and reported different dose-dependent levels of inactivation after phage treatment applied at 10^11^ and 10^9^ PFU/ml; corresponding inactivation rates observed were 2.72 log_10_ CFU/g and 1.47 log_10_ CFU/g, respectively. Islam et al. (2019) evaluated a cocktail of three new *Salmonella* phages (LPSTLL, LPST94, and LPST153) applied at concentrations of 10^7^ and 10^6^ PFU/ml for up to 24H at 25°C and 4°C; irrespective to the phage dose use, complete inactivation was consistently observed after 3 h of exposure in all cases. Bao et al. (2015) evaluated a mix of two *Salmonella* phages (PA13076 and PC2184) applied at 4°C and 25°C and reported 1.65 log_10_ CFU reduction after 1H of treatment. Spricigo et al. (2013) reported successful bacterial inactivation achieved by a mix 3 bacteriophages (UAB_Phi20, UAB_Phi78, and UAB_Phi87) applied up to 7 days at 4°C against *S a*. Typhimurium ATCC14028 (UA1872) and *Sa. Enteritidis* LK5 (UA1894); bacterial reductions of the two strains tested in parallel were 2.2 log_10_ CFU/g and 1.4 log_10_ CFU/g, respectively. Sofy et al. (2020) tested the antimicrobial activity of the new phage CoNShP-3 applied against Coagulase-negative *Staphylococcus* spp. at 4°C and −20°C; the phage treatment applied for 7 days at refrigeration temperature achieved 5.18 log_10_ CFU reduction; lower levels of inactivation were observed when the treatment was applied at lower temperature (see Table 2).

A smaller level of bacterial removal was also documented by studies using commercially available phage products. Sukumaran et al. (2015) used the commercial SalmoFresh^TM^ applied at different concentrations of phage (10^9^ and 10^8^ PFU/ml) against a mix of *Salmonella* spp; irrespective to the phage dose used, the study reported a reduction of the bacterial population of ∼1 log_10_ CFU after 1 and 7 days post-phage treatment. Yang et al. (2017) assessed antimicrobial abilities of the commercial listeriophage product ListShield^TM^ applied at 4°C for different times of incubation; inactivation rates observed after 1H, 24H, 48H, and 72H were 0.56, 0.84, 0.46, and 0.10 log_10_ CFU/g, respectively.

Two additional studies assessed the phage-based inactivation in mixed diced chicken meat purchased from local supermarkets. Thung et al. (2019) used a newly isolated CJ01 phage and reported 1.37 log_10_ CFU and 1.68 log_10_ CFU reduction of *C. jejunii* after 24H and 48H post-phage incubation at 4°C. Thung et al. (2017) tested the phage SE07 against *Sa. enterica* serovar Enteritidis and observed increasing level of bacterial inactivation (1.82. 1.95, and 2.02 log_10_ CFU/g) after 12H, 24H, and 48H of post-phage incubation at 4°C.

Evidence of bacterial reduction in chicken and turkey minced meat is also available in the scientific literature assessed. Yeh et al. (2017) applied the commercial Salmonelex^TM^ against a cocktail of 3 *Salmonella* spp. in ground chicken and ground turkey; bacterial population was reduced by 1.1 log_10_ CFU/g in ground chicken and 0.9 log_10_ CFU/g in ground turkey.

Five studies tested the phage-based removal of *Salmonella* spp. in eggshell (Spricigo et al. 2013, Thung et al. 2017, Ashrafudoulla et al. 2023) and liquid eggs or egg yolk (Guenther et al. 2012, Duc et al. 2020). Ashrafudoulla et al. (2023) tested the antimicrobial removal of a mix of two phages applied at different concentration (10^10^, 10^8^, and 10^7^ PFU/ml) against *Salmonella* serovar Thompson after 7 days at 20°C and 10°C; the highest level of bacterial reduction (2.5 log_10_ CFU/cm^2^) was observed after 7 days at 10°C. Similarly, Thung et al. (2017) evaluated the bacterial inactivation of the phage SE07 against *Sa. enterica* serovar Enteritidis; inactivation rates after 12H, 24H, and 48H post-phage incubation at 4°C were 1.7, 1.84, and 1.89 log_10_ CFU/cm^2^, respectively. Spricigo et al. (2013) used a mix of two phage (UAB_Phi20, UAB_Phi78, and UAB_Phi87) against two strains of *Salmonella* spp. (*Sa*. Typhimurium ATCC14028 (UA1872) and *Sa*. Enteritidis LK5 (UA1894)) on eggshell; level of bacterial reduction observed on broken eggs compared to control was 0.9 log_10_ CFU/g in both cases. Significant reduction of the bacterial population was also reported by two studies that involved phage treatment applied on directly to liquid eggs: Duc et al. (2020) reported ∼2 log_10_ after 2H at 24°C. In contrast, no significant reduction was reported by Guenther et al. (2012) who tested the phage F01-E2 against of *Sa*. Typhimurium in egg yolk.

### Raw seafood products

Due to the high perishable nature, freezing is often the main process applied to fish and fish products techniques to prevent food safety issues and ensure economic benefits for the fish companies. However, due to its nutritious nature and the overgrowing demand over the last past decades, increasing interest has been put on new and mild technologies able to maintain quality of food similar to what it is during its harvesting (Taveres et al. 2021). Considering this, bacteriophages appear a suitable solution for biocontrol of pathogens in sea food. Studies carried out by Zhang et al. (2018) and Jun et al. (2014) reported successful bacterial inactivation (99% inactivation and ∼6 log_10_ CFU/g) of *V. parahaemolyticus* in oysters stored 72H post-phage treatment at 15°C and room temperature in ice cubes. Similarly, Guenther et al. (2012) evaluated the phage FO1-E2 against *Sa*. Typhimurium in mixed seafood and observed complete inactivation after 24H at 8°C and 1 log_10_ CFU reduction after 2 days at 15°C. Two additional studies evaluated bacterial inactivation of *L. monocytogens* in catfish fillets and salmon fillets treated with the commercial listeriophage product Listex™P100. Soni and Nannapaneni (2010a) used Listex™P100 at different phage concentrations to evaluate the listericidal effect on small (5 g) and big catfish fillets (180–220 g) at 4, 10 and 22°C; the level of bacterial inactivation observed ranged 0.8 to 2.1 log_10_ CFU/g, depending on different time/temperature combinations applied (see table 2). Soni and Nannapanemi (2010b) attempted to optimize removal rates of Listex™P100 in salmon filles spiked at different levels with *Listeria monocytogenes* then stored at 4 and 22°C after phage treatment: higher phage concentrations proved to be more effective and demonstrated significantly higher bacterial reductions at either 4°C and 22°C (Table 2)

### Ready-to-eat food

A growing body of scientific publications assessed the biocontrol of pathogens in different ready-to-eat (RTE) food including dry-cured ham, sliced fish and meat products, hotdog and soft cheeses.

You et al. (2021) described significant removal of *V. parahaemolyticus* in RTE sliced fish treated with phage VPT02 after 6H at 25°C and 5°C. Similarly, three studies carried out by Iacumin et al. (2016), Guenther et al. (2012), and Bigot et al. (2011) described successful inactivation of pathogens in sliced animal food products and hotdogs. Iacumin et al. (2016) optimized phage-based conditions to maximize removal of *L. monocytogenes* in dry-cured ham slices at 4°C, 10°C, and 24°C; among different phage/cells ratios considered, Listex P100^TM^ used at concentration of 10^8^ PFU/ml proved to be the optimal condition and achieved complete bacterial inactivation after 24H at each of the temperature of storage considered. Guenther et al. (2012) and Bigot et al. (2011) demonstrated significant bacterial removal in sliced poultry products and hotdogs. Guenther et al. (2012) observed completed inactivation in sliced chicken and hotdog spiked with *Sa*. Typhimurium then treated with phage F01-E2; Bigot et al. (2011) tested a new phage isolates FWLLm1 against *L. monocytogenes* in sliced chicken breast; the phage treatment was able to reduce listeria population by 2.5 and 1.5 log_10_ CFU after 48H at 30°C and 7 days at 5°C, respectively.

Finally, two additional studies described phage-based bacterial inactivation of *Listeria monocytogenes* in soft cheeses. Silva et al. (2014) compared antibacterial activity of the commercially available Listex™P100 applied at 21°C and 4°C in Minas Frescal and Coalho cheese spiked with 4 log_10_ CFU/g of *L. monocytogenes;* higher levels bacterial inactivation were generally observed for phage treatments applied at 21°C (2.3 and 2.1 log_10_ CFU/g) rather than at 4°C (1 log_10_ and 0.8 log_10_ CFU observed for the two cheeses, respectively); Guenther and Loessner (2011) attempted to optimize phage-based conditions for biocontrol on *L. monocytogenes* in white-mold cheese spiked at high and low levels of bacterial load; with lower level of listeria contamination (10–10^2^ CFU/cm^2^), viable counts dropped below the limit of detection after 6 days at 6°C; in contrast, biocontrol of higher bacterial contamination achieved lower removal corresponding to 2 log_10_ CFU/g for sample treated with a single dose, and 3 log_10_ CFU/g after 6 days for samples treated at days 1 and 6.

### Biocontrol of pathogens in vegetables and sliced fruit

Thirteen of the selected studies focused on biocontrol of pathogens in vegetables including cherry tomatoes (*n* = 1), lettuce (*n* = 8), broccoli (*n* = 1), fruit (*n* = 1), and fruit slices (*n* = 1). El-Doughdoug et al. (2019) demonstrated significant reduction of *Salmonella* serovar Newport adhering cherry tomatoes.

Eight studies documented successful application of bacteriophages applied to lettuce for biocontrol of *St. aureus* (Lambuk et al. 2024), *Salmonella* spp. (Spricigo et al. 2013, Sadekuzzaman et al. 2018, Duc et al. 2020), pathogenic *E. coli* (Ferguson et al. 2013, Lu et al. 2022), and *L. monocytogenes* (Sadekuzzaman et al. 2017b). Lambuk et al. (2024) described the optimization of phage-based treatment based on phage mSA4 against *St. aureus* in iceberg lettuce and reported 3.2 log_10_ CFU inactivation after 24H at 4°C. Duc et al. (2020) applied the polyvalent phage PS5 against *Sa*. Enteritidis, *Sa*. Typhimurium, and *E. coli* O157: H7 and observed complete inactivation of the two pathogens after 2H at 4°C and 24°C. Similarly, Sadekuzzaman et al. (2018) described significant bacterial reduction (>1 log_10_/CFU) achieved by a mix of phages (BP1370 and BP1369) applied against *Sa. enterica* serovars for 2H at 10°C, 20°C, and 25°C. Spricigo et al. (2013) applied a mix of three phages (UAB_Phi20, UAB_Phi78, and UAB_Phi87) against two *Salmonella* spp. strains [*Sa*. Typhimurium ATCC14028 (UA1872) and *Sa*. Enteritidis LK5 (UA1894)]; inactivation rates observed after 1H at room temperature ranged 1.9–3.9 log_10_ CFU. Lu et al. (2022) tested three phages, VE04, VE05, and VE07, against different strains of *E. coli* O157: H7; although variations between bacteriophages and strains used, the study demonstrated significant reduction of the bacterial population with inactivation rates ranging 2.6–6 log_10_ CFU. Sadekuzzaman et al. (2017b) used phage BPECP 19 applied at 10°C, 20°C, and 25°C for biocontrol of *E. coli* O157: H7; significant reduction (>2 log_10_/CFU) was observed in all cases. Ferguson et al. (2013) tested the commercially available EcoShield™ against *E. coli* O157: H7 and reported a reduction of 1.1 log_10_ CFU after 24H at 4°C. Similarly, Sadekuzzaman et al. (2017a) documented 0.7 log_10_ CFU reduction achieved by the commercially available ListSheild™ applied for 2H at 10°C, 20°C, and 25°C against *L. monocytogenes*.

Additional range of produce, including celery, broccoli, cantaloupe, strawberries, and sliced-fruit have been considered in recent years during phage-based inactivation studies. Byun et al. (2024) applied a cocktail of phages (LMPC01, LMPC02, LMPC03) for biocontrol of *Listeria monocytogenes* and reported 1.63 log_10_ CFU inactivation after 5 days a 4°C. Magnone et al. (2013) tested three commercial bacteriophage products (EcoShield^TM^, SalmoFresh^TM^, and ShigActive^TM^) for biocontrol of *E. coli* O157: H7, *Sa*. spp., and *Shigella* spp. in broccoli, cantaloupe, and strawberries; SalmoFresh^TM^ achieved the highest level of inactivation against *Salmonella* spp. in broccoli (2.79 log_10_ CFU); a levels of inactivation of ∼0.34–0.78 log_10_ CFU were observed in all the other cases. Oliveira et al. (2014) evaluated Listex™P100 for biocontrol of *Listeria* spp. in melon, pear, and apple slices; the product achieved 1.5 log_10_ and 0.6 log_10_ reduction in melon and pear slices; in contrast, no significant bacterial reduction was observed for apple slices treated in parallel.

### Biocontrol of pathogens in liquid food and drinks

Alongside to biocontrol studies focusing on abiotic and biotic surfaces, additional eight studies providing evidence of penetration power and inactivation ability of bacteriophages in liquid food and drinks were selected for this review.

Three studies assessed biocontrol of pathogens in milk: Song et al. (2021) reported 95% reduction of *St. aureus* in milk treated in phage Vb-SAV-SDQ; Duc et al. (2020) assessed inactivation rates of polyvalent phage PS5 against *Sa*. Enteritidis, *Sa*. Typhimurium, and *E. coli* O157: H7 and observed ∼2 log_10_ CFU reduction after 2H post-phage treatment applied at 4°C and 24°C; Bao et al. (2015) used a mix of two phages (PA13076 and PC2184) against *Sa*. Enteritidis in whole pasteurized milk and described 3.89 log_10_ of bacterial reduction after 1H at 4°C and complete inactivation after 1H at 25°C.

Two more studies attempted to evaluate biocontrol of pathogens in chocolate milk: Lambuk et al. (2024) obtained 2.1 log_10_ CFU inactivation of *St. aureus* treated phage PA13076 after 24H at 4°C; Guenther et al. (2012) reported completed inactivation of *Sa*. Typhimurium in phage-treated chocolate milk at 8°C and 15°C.

Efficacy of phages for biocontrol of pathogens in fruit and energy drinks was finally evaluated in 2 selected studies. Thung et al. (2017) reported significant reduction (2–2.2 log_10_/CFU) of *Sa. enteterica serovar Enteritidis* after 12H, 24H, and 48H at 4°C in phage-treated fruit juice. Conversely, different levels of bacterial inactivation were observed by Oliveira et al. (2014) in melon juice, pear juice, and apple juice treated with Listex™P100; despite high inactivation rates (3 log_10_ CFU) achieved in pear juice after 8 days at 4°C, lower level of bacterial reduction of listeria population (0.6 log_10_ CFU) was observed in melon juice; not significant bacteria reduction was observed in apple juice treated in parallel.

### Stability under food processing conditions

Nineteen of the 50 selected studies investigated the stability of phages under food processing conditions, including pH (*n* = 14) and thermal fluctuations (*n* = 15) as well as their stability in food matrices (*n* = 4). A brief description of findings is provided below in reverse chronological as used in Table 3.

**Table 3.** Summary of results regarding thermal stability, pH stability, and stability in food matrices.

Bacteriophage identification	Thermal stability	pH stability	Stability in food	References
mSA4	4°C–37°C > 1H >4 log_10_ reduction at 65°C	pH 4–11 inactivated at pH 3 and 12	N/A	Lambuk et al. (2024)
CAU-STP-1, CAU-STP-2	Stable at 60°C 2 log_10_ reduction at 65°C 4 log_10_ reduction at 70°C	pH 4–11 pH 5–11	N/A	Ashrafudoulla et al. (2023)
SEPL01, SEPL13, SEPL20	Stable at 4°C–7°C > 1 h 1 log_10_ reduction at 50°C and 60°C 2.5 log_10_ reduction at 70°C	pH 4–11 >4 log_10_ reduction at pH3, 11 and 12	N/A	Kokab et al. (2022)
VE04, VE05, VE07	$- $20°C–37°C for 31 days	pH 6–10 inactivated at pH 2 and 4	N/A	Lu et al. (2022)
Ace, Shy	N/A	pH 4–11 complete inactivation at pH 1 and 13	N/A	Pinto et al. (2021)
VPT02	4°C–50°C for 12H Inactivated at 60°C and 70°C	pH 3–11 inactivated at pH 2 and 12	N/A	You et al. (2021)
Polyvalent phage PS5	40°C–60°C for 30 min Complete inactivation at 80°C	pH 4–10 for 1H complete inactivation at pH 2 and 11	N/A	Duc et al. (2020)
CoNShP-3	$-$20°C–50°C for 30 days Inactivated at 60°C and 70°C	pH 7–9 for 30 days inactivated at pH 4 and 11	N/A	Sofy et al. (2020)
CGG3-1, CGG3-2, CGG4-1, CGG4-2	−25°C–37°C >24 h Inactivated at 55°C	Stable at pH 5–9 for 24 h	N/A	El-Dougdoug et al. (2019)
CJ01	25°C, 37°C, and 42°C for 1H 4 log_10_ reduction at 4 and 55°C	pH 3–11 for 1H inactivated at pH 3	N/A	Thung et al. (2019)
BP1369, BP1370	4°C–50°C >180 min Inactivated at 70°C and 80°C	N/A	N/A	Sadekuzzaman et al. (2018)
vB_VpaS_OMN	30°C–50°C for 1H inactivation at > 60°C	pH 5–9 for 1H inactivated at pH 3, 4 and 4	N/A	Zhang et al. (2018)
BPECO 19	4°C–0°C > 180 min Inactivated at 60°C, 70°C, and 80°C	N/A	N/A	Sadekuzzaman et al. (2017b)
SE07	28°C, 37°C, and 42°C for 1H 5log_10_ reduction at 65°C	pH 4–11 for 1H inactivated at pH 3	N/A	Thung et al. (2017)
Listex™P100	N/A	N/A	Dry ham: stable 14 days at 4°C	Iacumin et al. (2016)
PA13076 and PC2184	30°C–50°C for 1H 2 log_10_ reduction at 60°C and 70°C Complete inactivation at 80°C and 90°C	pH 5–11 for 2H inactivated at pH 3, 4, 12, and 13	Chicken breast: Stable 24H at 4°C and 25°C 1–2 log_10_ reduction after 48H and 72H Milk: stable 72H at 4°C and 25°C Chinese cabbage: Stable 5H at 4°C and 25°C 1–3 log_10_ reduction after 24 and 72H	Bao et al. (2015)
Φ534, Φ557	4°C–37°C >7 days >2 log_10_ reduction at 60°C and 68°C	pH 7 and 9 for 28 days inactivated at pH 3 after 3 days	N/A	Allué-Guardia et al. (2014)
Listex™P100	N/A	N/A	Catfish: 1 log_10_ reduction after 24H at 4°C; >2 log_10_ reduction after 4 days	Soni et al. (2010)
Listex™P100	N/A	N/A	Salmon fillets: Stable 4 days at 4°C, 0.6 log_10_ reduction after 10 days	Song and Nannapaneni et al. (2010b)

N/A, information not available.

Results provided by many authors are consistent that bacteriophages can remain stable within a broad range pH varying from 4 to 11. Lower and higher pH conditions were consistently reported to affect infectivity and reduce the phage titre (Thung et al. 2017, Thung et al. 2019, Duc et al. 2020, Pinto et al. 2021, You et al. 2021, Kokab et al. 2022, Ashrafudoulla et al. 2023, Lambuk et al. 2024). Narrower pH tolerance is reported by Bao et al. (2015), Zhang et al. (2018), Sofy et al. (2020), Lu et al. (2022), and Allué-Guardia et al. (2014).

Many studies also investigated stability of bacteriophages at storage and food preparation temperatures. Sadekuzzaman et al. (2018), Sadekuzzaman et al. (2017b), Duc et al. (2020), and You et al. (2021) demonstrated the stability of phages at temperatures ranging 4°C–50°C. Two additional studies including Lu et al. (2022) and Sofy et al. (2020) also reported the ability to survive at lower temperature but there seems to be no evidence about their real ability to be used as biocontrol agents for food frozen at −20°C or −25°C. High temperatures proved to have a detrimental impact on phage biology: six studies documented rapid phage inactivation at temperatures higher than 60°C (Thung et al. 2017, Sadekuzzaman et al. 2017b, Zhang et al. 2018, Thung et al. 2019, You et al. 2021, Kokab et al. 2022)

Four studies also investigated the survival rates of phages in food matrices. Iacumin et al. (2016) reported the stability of the bacteriophage products Listex™P100 in dry ham up to 14 days at 4°C; Similarly, Song and Nannapaneni (2010b) reported similar stability rates of the same bacteriophage product in salmon filets; Bao et al. (2015) reported prolonged resistance of PA13076 and PC2184 in milk and chicken breast at 4°C and 25°C. Conversely, limited resistance is reported by Soni and Nannapaneni (2010a) in catfish at 4°C. A detailed description of findings from studies included in this section is reported in Table 3.

### Bacterial re-growth after phage treatment

Eight of 50 studies included in this review reported bacterial regrowth in food (7) and food-contact surfaces (1) after bacteriophage treatment (Table 4). Siringan et al. (2011) tested efficacy phage CP30 against biofilms of *C. jejuni* NTCT11168 grown up to 24H at 37°C on glass; the study reported a rapid increase of the viable count 2 h post phage treatment; Duc et al. (2020) observed post-phage bacterial regrowth after 6H in beef, chicken skin, pasteurized milk and liquid egg treated with the polyvalent phage PS5 24H at 24°C; Byun et al. (2024) tested the efficacy of a phage cocktail containing phages LMPC01, LMPC02, LMPC03 against *L. monocytogenes* in celery stored 7 days at 4°C; significant increase of bacterial population was observed after initial 1.63 log_10_ bacterial reduction achieved during the first 5 days of food storage applied after phage treatment. Yang et al. (2017) assessed the bacterial inactivation of commercial ListSheild™ applied alone and combined to UV treatment at 600, 1800, and 2400 mWs/cm^2^ in with listeria*-*spiked chicken breast store pup to 72H at 4°C; despite initial bacterial reduction observed after 1H post post-phage treatment, increase in the viable count was consistently observed for the all the combined phage-based treatments applied in parallel. Bao et al. (2015) used phages PA13076 and PC2184 against *Sa*. Enteritidis in chicken breast stored 5 h at 4°C and 25°C; after initial 1.65 log_10_ reduction 1H post phage treatment, increase of the viable counts was reported for all the samples stored at 25°C. Guenther et al. (2012) tested the antimicrobial efficacy of the phage F01-E2 against *Sa*. Typhimurium in different food including hotdog, sliced turkey, mixed seafood, egg yolk, and chocolate milk; although significant inactivation observed 2 days after phage treatment, consistent bacterial regrowth was observed during the remaining 4 days of storage at 15°C. Bigot et al. (2011) used phage FWLLm1 against *L. monocytogenes* in chicken breast stored 25 days at 30°C and reported bacterial regrowth after initial 2.5 log_10_ reduction 48H post-phage treatment. Guenther and Loessner (2011) attempted to optimize phage-based conditions to maximize inactivation of *L. monocytogens* in White mold cheese and Red-smear cheese; although promising inactivation rates observed after 6 days at 6°C, a level of bacterial regrowth was observed during the remaining 22-day food storage applied after phage treatment.

**Table 4.** Summary of findings from studies reporting evidence of bacterial regrowth after phage treatment applied to food and food-contact surfaces.

Host	Bacteriophage	Titre (PFU/ml)	MOI	Food/food contact material	Storage post-phage treatment (PPT)	Description of findings	Reference
*Campylobacter jejuni* NCTC 11168	CP30	10^9^ PFU 10^6^ PFU	10^3^ 10	Glass	24H at 37°C	bacterial regrowth 2H PPT	Siringan et al. (2011)
*Salmonella* Enteritidis, *Salmonella* Typhimurium, and *Escherichia coli* O157:H7	Polyvalent phage PS5	10^8^ PFU	10^4^ 10^3^	Beef Chicken skin Pasteurized milk Liquid eggs	24H at 24°C	Bacterial regrowth after initial 3.5 log_10_ reduction 6H PPT Bacterial regrowth after initial 2.4 log_10_ reduction 6H PPT Bacterial regrowth after initial ∼2 log_10_ reduction 2H PPT Bacterial regrowth after initial ∼2 log_10_ 2H PFT	Duc et al. (2020)
*Listeria monocytogenes*	Phage cocktail (LMPC01, LMPC02, LMPC03 + NaOH	10^8^ PFU	10^4^	Celery	7 days 4°C	Bacterial regrowth after initial 1.63 log_10_ CFU 5 days PPT	Byun et al. (2024)
*Listeria monocytogenes*	Commercial ListSheild™ + UV treatment appled at 600, 1800 and 2400 mWs/cm^2^	10^8^ PFU	10^3^	Chicken breast	72H at 4°C	Bacterial regrowth after initial 1.5–2.05 log_10_ reduction 1H PPT	Yang et al. (2017)
*Salmonella* Enteritidis	PA13076 and PC2184 (isolated from chicken sewage—mix)	10^9^ PFU	10^4^	Chicken breast	5H at 4°C 5H at 25°C	No regrowth observed PFT Bacterial regrowth after initial 1.65 log_10_ reduction 1H PPT at 25°C	Bao et al. (2015)
*Salmonella* Typhimurium	FO1-E2	10^8^ PFU	10^4^	Hotdog Sliced turkey Mixed seafood Egg yolk Chocolate milk	6 days at 15°C	Bacterial regrowth after initial complete inactivation after 2 days PPT Bacterial regrowth after initial complete inactivation after 2 days PPT Bacterial regrowth after initial 1 log_10_ reduction 2 days PPT Bacterial regrowth 2 days PPT Bacterial regrowth after initial 1 log_10_ reduction 2 days PPT	Guenther et al. (2012)
*Listeria monocytogenes*	FWLLm1 (isolated from sheep faeces)	10^7^ PFU	10^2^	Sliced chicken breast	25 days at 30°C	Bacterial regrowth after initial 2.5 log_10_ reduction 48H PPT	Bigot et al. (2011)
*Listeria monocytogenes*	A511 alone (white mold cheese) and A511 added to brine + 3% NaCl and LAB	10^9^ PFU 10^8^ PFU	10^8^ 10^7^ 10^5^ 10^4^	White-mold cheese Red-smear cheese	22 days at 6°C	Bacterial regrowth after initial 2–3 log_10_ reductions 6 days PPT Bacterial regrowth after initial 2–3 log_10_ reductions 6 days PPT	Guenther and Loessner (2011)

PPT, post phage treatment.

### Synergistic effect of phage-based treatments applied to food and food-contact surfaces

Six studies conducted a comparative analysis about the effectiveness of bacteriophages applied alone and combined with additional chemical or physical treatments. Results of the studies are summarized in Table 5.

**Table 5.** Summary of combined phage-based treatments assessed for biocontrol of pathogens in food and food-contact surfaces.

Host	Bacteriophage	Titre (PFU/mL)	Cofactor tested	Treatment	Matrix	Reduction log10 CFU/cm^2^	References
*Listeria monocytogenes*	Phage cocktail (LMPC01, LMPC02, LMPC03	10^8^ PFU	185 ppm NaOH	3H at 4°C 6H at 4°C	Polyethylene (PE)	Phage alone: 1.04 log_10_ after 3 h 1.59 log_10_ after 6 h Phage-NaCl 2.2 log_10_ after 3 h 4.89 log_10_ after 6 h	Byun et al. (2024)
					Polypropylene (PP)	Phage alone: 1.06 log_10_ after 3 h 1.69 log_10_ after 6 h Phage-NaCl 2.59 log_10_ after 3 h 4.56 log_10_ after 6 h	
					Stainless steel (SS)	Phage alone: 1.16 log_10_ after 3 h 1.76 log_10_ after 6 h Phage-NaCl 2.59 log_10_ after 3 h 4.42 log_10_ after 6 h	
*Escherichia coli* O157:H7,	Commercial EcoShield (bc)	10^10^ PFU	Levulinic acid produce wash (PW)	24H at 10°C	Strawbery Cantaloupe Broccoli	bc: 0.53 log_10_ BCPW: Below the limit of detection bc: 0.74 log_10_ BCPW: Below the limit of detection bc: 0.68 log_10_ BCPW: Below the limit of detection	Magnone et al. (2013)
*Salmonella spp*,	Commercial The SalmoFresh (bc)	10^10^ PFU	Levulinic acid produce wash (PW)	24H at 10°C	Strawbery Cantaloupe Broccoli	bc: 0.53 log_10_ BCPW: Below the limit of detection bc: 0.78 log_10_ BCPW: 2.96 log_10_ bc: 2.79 log_10_ BCPW: 4.55 log_10_	
*Shigella* spp	Commercail ShigActive (bc)	10^10^ PFU	Levulinic acid produce wash (PW)	24H at 10°C	Strawbery Cantaloupe	bc: 0.34 log_10_ BCPW: Below the limit of detection bc: 0.75 log_10_ BCPW: Below the limit of detection	
					Broccoli	bc: 0.32 log_10_ BCPW: Below the limit of detection	
*Listeria monocytogenes*	Commercial ListSheild™ (PA)	10^8^ PFU	UV-C light	1H 24H at 4°C 48H at 4°C 72H at 4°C	Chicken breast	PA: 0.56 log_10_ after 1H 0.84 log_10_ after 24H 0.46 log_10_ after 48H 0.10 log_10_ after 72H Combined Phage-UV-C light: Phage + 600 mW/cm^2^: 1.5 log_10_ after 1H Phage + 1200 mW/cm^2^: 1.5 log_10_ after 1H Phage + 1800 mW/cm^2^: 1.5 log_10_ after 1H Phage + 2400 mW/cm^2^: 2.05 log_10_ after 1H	Yang et al. (2017)
*S almonella* Typhimurium *Salmonella*, Heidelberg, and *Salmonella* Enteritidis	SalmoFresh™ (PA)	10^9^ PFU 10^9^ PFU	HCL, CPC, LAE, PAA	24H at 4°C	Chicken skin	PA: 1 log_10_ Chlorine + phage: 1.8 log_10_ LAE + phage: 1 log_10_ PAA 50 ppm + phage: 2.2 log_10_ PAA 400 ppm + phage: 2.5 log_10_	Sukumaran et al. (2015)
			HCL, CPC, LAE, PAA	7 days at 4°C	Chicken breast	PA: 1.1 log_10_ after 1 day PA: 1 log_10_ after 7 days Phage + 0.6% CPC: 1.2 log_10_ after 1 day Phage + 0.6% CPC: 1.4 log_10_ after 7 days Phage + 200 ppm LAE: 1 log_10_ after 1 day Phage + 200 ppm LAE: 1 log_10_ after 7 days	
		10^8^ PFU				PA: 1.1 log_10_ after 1 day PA: 1 log 10 after 7 day Phage + 0.6% CPC: 0.9 log_10_ after 1 day Phage + 0.6% CPC: 1 log_10_ after 7 days Phage + 200 ppm LAE: 1 log_10_ after 1 day	
						Phage + 200 ppm LAE: 0.9 log_10_ after 7 days	
*Escherichia coli* O157:H7	Commercial EcoShield™ (PA)	10^9^ PFU	50 ppm HCl	24H at 4°C	Lettuce	PA: 1.1log_10_ 50 ppm HCl: 0.9log_10_ 50 ppm HCl + phage: 1.9 log_10_	Ferguson et al. (2013)

Byun et al. (2024) applied a combination of LMPC01, LMPC02, and LMPC03 phages used alone or combined to NaCl, H_2_O_2_, lactic acid against *L. monocytogenes* grown on PP, PE, and stainless-steel surfaces; the phage cocktail applied combined to NaCl consistently achieved higher level of inactivation than phage treatment applied alone. Similarly, Magnone et al. (2013) evaluated the efficacy of three commercial bacteriophage products used alone and combined to levulinic acid produce wash against *E. coli* O157: H7, *Salmonella* spp. and *Shigella* spp. in strawberry cantaloupe and broccoli; combined treatment significantly increased bacterial removal in all cases. Yang et al. (2017) compared the effectiveness of the commercial ListSheild^TM^ applied alone and combined to UV treatment applied at 600, 1200, 1800, and 2400 mW/cm^2^ for biocontrol of *L. monocytogenes* in chicken breast; phage treatment followed by UV at 2400 mW/cm^2^ was found to obtain the higher bacterial inactivation rate. Sukumaran et al. (2015) attempted to optimise inactivation rates of the commercial SalmoFresh™ used against *S*a. Typhimurium, *Sa*. Heidelberg, and *S*a. Enteritidis in chicken skin and chicken breast; among different combinations tested in parallel, high concentration of phage treatment combined to 400 ppm PAA and 0.6% CPC were found to be the most successful biocontrol solution for biocontrol of *Salmonella* spp. in chicken skin and chicken breast, respectively. Finally, Ferguson et al. (2013) compared the antimicrobial activity of the commercial EcoShield™ used alone and combined to 50 ppm HCl against *E. coli* O157: H7; the combined treatment achieved significantly higher inactivation rates compared to all the other conditions applied in parallel.

## Discussion

Foodborne disease linked to pathogenic biofilms still represents a relevant food safety issue and a big threat for the food industry (Abee et al. 2011, Liu et al. 2023, Khan et al. 2023). Biofilms are complex bacterial communities more resistant than planktonic cells to antibiotics, disinfectants, and sanitizing agents (Folsom and Frank 2006, Abee et al. 2011) and their control require alternative and more sustainable strategies. Due to their high host specificity bacteriophages are considered a great weapon against pathogens in food production settings because of completely harmless to people (Meaden and Koskella 2013, Kasman and Porter 2024) and due to their ability to not to affect sensory properties of food (Aprea et al. 2018, Moye et al. 2018). Since the first phage product received designation as *generally recognized as safe*Kawaka (GRAS) for direct application to food by the Food and Drug Administration (FDA), more than 25 bacteriophage products produced with different brand names have become commercially available during the last 2 decades (Kawacka et al. 2020). However, despite many successful trials in different food production settings, the large-scale of bacteriophages as biocontrol agents still represents a controversial topic (EFSA 2016, FDA 2024a, 2024b, 2024c) and their usage seems to be allowed only in some countries, including USA, Australia, New Zealand, Israel, Canada, Switzerland, and Netherlands (Kawacka et al. 2020). One of the main concerns is that widespread use of these bacteriophage treatments might potentially promote the emergence of phage resistant mutants (Moye et al. 2018).

In order to overcome these issues and minimize all the associated risks, substantial amount of research has been carried out to define relevant characteristics for phage’s to be used in food production settings. To be safely used in food and food processing environments ideal phages should meet key specific requirements: (i) being strictly lytic phage in order to avoid lysogenic conversion inside the host; (ii) having broad host-range in order to kill as many target strains as possible; (iii) inability to transfer genetic material including virulence factors from one host to another; (iv) absence of genes encoding for pathogenicity factors and allergenic proteins; (v) sufficient stability under food preparation conditions, including pH, thermal fluctuation, and stability in food matrices; and (vi) ability to propagate also in a non-pathogenic host to avoid large-scale production pathogen cultures (Cristobal-Cueto et al. 2021, Garvey 2022, Kawacka et al. 2020). As recently reviewed by Kawacka et al. (2020) specific measures to minimize risk of phage resistance in phage-treated food have been also identified. These include: (i) not re-entering the products already treated with phages into production cycle; (ii) administrating phage treatment to products immediately before packaging or shipment to avoid the contact between treating phages and bacterial flora normally occurring in food preparation settings; (iii) avoidance of re-inoculation techniques such as ‘young-old smearing,’ as applied in some cheese making technologies and consisting of re-using part of bacterial flora from mature cheeses to wash young cheeses; (iv) use of mixture/cocktail products containing more than one phage with different host specificity to minimize the risk of resistance towards individual phages (Moye et al. 2018; Kawacka et al. 2020). In this systematic review, we evaluated the most recent scientific information made available over the last 14 years on the efficacy of phage-based preparations in relation to their use in food production settings. Analysis of the literature on the efficacy of bacteriophages as biocontrol agents in food and food-contact surfaces seems to describe two different pictures.

Despite the existence of differing results and diverse experimental conditions, most of the studies selected confirm the efficacy of bacteriophages as bio-sanitizing against pathogenic biofilms in food contact materials. In 13 out 14 studies focusing of food contact materials, either commercial preparations (Soni and Nannapaneni 2010a, Montanez-Izquierdo et al. 2012, Iacumin et al. 2016, Gutiérrez et al. 2017, Sadekuzzaman et al. 2017a) and new phage isolates (Arachchi et al. 2013, Sadekuzzaman et al. 2017b, 2018, Islam et al. 2019, Wang et al. 2020, González-Gómez et al. 2021, Byun et al. 2024) achieved significant viable bacterial reduction and demonstrated stability at temperatures and pH ranges commonly encountered in food processing environments; bacteriophages used also showed tolerance to diverse physical and chemical treatments (Allué-Guardia et al. 2014, Byun et al. 2024) and showed additional biofilm removal when used combined to disinfectants (Byun et al. 2024). Only one study conducted by Siringan et al. (2011) reported differing level of inactivation achieved by two phage isolates (CP8 and CP30) against two *Campylobacter* spp. strains (NCTC 11168 and PT14) and potential resistance of *C. jejuni* NCTC 11168 against phage CP30. Apart from this, the overall picture emerging from the analysed literature seems to confirm the overall ability of bacteriophage to remove bacterial biofilms from all the surfaces tested with different levels of reduction varying from ∼1 log_10_ (Sadekuzzaman et al. 2017a, Byun et al. 2024) to 2–5 log_10_ (Soni and Nannapanemi 2010a, Montañez-Izquierdo et al. 2012, Arachchi et al. 2013, Iacumin et al. 2016, Gutiérrez et al. 2017, Sadekuzzaman et al. 2017b, 2018, Islam et al. 2019, Wang et al. 2020, González-Gómez et al. 2021). Use of cocktails as applied in some comparative studies also demonstrated to be slightly more effective than individual phages (Arachchi et al. 2013, Jaroni et al. 2023).

Conversely, existence of conflicting reports, different methodologies and food storage conditions used make the interpretation of findings about the efficacy of bacteriophages for biocontrol of pathogens in food a little more difficult. Despite the existence of differing results, the scientific evidence seems to confirm the ability of bacteriophages to achieved significant levels of inactivation against different pathogens adhering a broad range of food tested, with higher level of inactivation generally observed in some comparative studies when the phage treatment is applied at mild temperature compared to refrigeration temperature (Soni and Nannapaneni 2010a, 2010b, Bigot et al. 2011, You et al. 2021) or lower. There seems to be some scientific evidence suggesting the ability of some phages to survive at freezing temperature (El-Doughdough et al. 2019, Sofy et al. 2020). However, not information seems to be available in the retrieved literature about their real ability to work as biocontrol agents in frozen food.

The phage-based treatment also demonstrated also significant penetration power and inactivation rates in liquid food and drinks (Guenther et al. 2012, Bao et al. 2015, Thung et al. 2017, Duc et al. 2020, Song et al. 2021, Lambuk et al. 2024). However, apart from very successful applications (Iacumin et al. 2016, Islam et al. 2019, Song et al. 2021) not all the studies reported complete bacterial inactivation as hoped. In parallel to initial reduction of the viable count, bacterial regrowth post-phage application was also documented in more than one study (Bigot et al. 2011, Guenther et al. 2012, Bao et al. 2015, Yang et al. 2017, Duc et al. 2020, Byun et al. 2024) even after repeated phage applications (Guenther and Loessner 2011).

To overcome these issues, additional efforts have been made by researchers to explore potential improvements to this technology, and the efficacy of bacteriophages used alone or combined to other physical and chemical treatment has also been explored. Findings reported seems to enhance as reported by Ferguson et al. (2013), Sukumaran et al. (2015), and Magnone et al. (2013), combined use of bacteriophages with other treatments gave clear proof of concept and proved to be more successful than phage treatment applied alone. Based on these findings, this route appears a more realistic option to fully meet needs of modern food production.

### Future perspectives

Foodborne illness caused by pathogenic bacteria adhering to food and food-contact surfaces still represents a big treat to the food industry. Due to high level of specificity for their host bacteria, bacteriophages have emerged as a promising potential alternative to antibiotics and disinfectants. Their stability under different environmental conditions as well stability in different food matrices make bacteriophages a suitable solution for biocontrol of pathogens in food production. However, current findings showed different level of robustness when this technology is applied to food or food-contact surfaces. Whether the use of bacteriophages can be completely accepted as a standalone treatment for food surfaces, their application to food shows higher level of inactivation achieved when bacteriophages are applied combined to other treatments rather than used alone. This seems to suggest that further optimizations are possibly still needed in order to maximize the efficacy of existing phage-based treatments for biocontrol of pathogens in food products and to minimize the risk of bacterial survival after treatment.

## Supplementary Material

qvaf005_Supplemental_File

## Data Availability

All findings reported in this review are available through the referenced original research citations. Data sharing is not applicable to this article as no new data are included created or analysed in this review.

## References

[bib1] Abee T, Kovács ÁT, OP K et al. Biofilm formation and dispersal in Gram-positive bacteria. Curr Opin Biotechnol. 2011;22:172–9. 10.1016/j.copbio.2010.10.016 21109420

[bib3] Allué-Guardia A, Martínez-Castillo A, Muniesa M. Persistence of infectious Shiga toxin-encoding bacteriophages after disinfection treatments. Appl Environ Microb. 2014;80:2142–9. 10.1128/AEM.04006-13 PMC3993145 24463973

[bib4] Aprea G, Zocchi L, Di Fabio M et al. The applications of bacteriophages and their lysins as biocontrol agents against the foodborne pathogens *Listeria monocytogenes* and *Campylobacter* spp.: an updated look. Vet Ital. 2018;54:293–303. 30681128 10.12834/VetIt.311.1215.2

[bib5] Arachchi GGJ, Cridge AG, Dias-Wanigasekera BM et al. Effectiveness of phages in the decontamination of *Listeria monocytogenes* adhered to clean stainless steel, stainless steel coated with fish protein, and as a biofilm. J Ind Microbiol Biotechnol. 2013;40:1105–16. 10.1007/s10295-013-1313-3 23907252

[bib6] Ashrafudoulla M, Kim HJ, Her E et al. Characterization of *Salmonella* Thompson-specific bacteriophages and their preventive application against *Salmonella* Thompson biofilm on eggshell as a promising antimicrobial agent in the food industry. Food Control. 2023;154:1–12. 10.1016/j.foodcont.2023.110008

[bib7] Bai X, Nakatsu CH, Bhunia AK. Bacterial biofilms and their implications in pathogenesis and food safety. Foods. 2021;10:2117. 10.3390/foods10092117 34574227 PMC8472614

[bib8] Ban GH, Yoon H, Kang DH. A comparison of saturated steam and superheated steam for inactivation of *Escherichia coli O157, H7, Salmonella typhimurium*, and *Listeria monocytogenes* biofilms on polyvinyl chloride and stainless steel. Food Control. 2014;40:344–50. 10.1016/j.foodcont.2013.12.017

[bib9] Bao H, Zhang P, Zhang H et al. Bio-control of *Salmonella Enteritidis* in foods using bacteriophages. Viruses. 2015;7:4836–53. 10.3390/v7082847 26305252 PMC4576208

[bib10] Bigot B, Lee WJ, McIntyre L et al. Control of *Listeria monocytogenes* growth in a ready-to-eat poultry product using a bacteriophage. Food Microbiol. 2011;28:1448–52. 10.1016/j.fm.2011.07.001 21925027

[bib11] Byun KH, Han SH, Choi MW et al. Efficacy of disinfectant and bacteriophage mixture against planktonic and biofilm state of *Listeria monocytogenes* to control in the food industry. Int J Food Microbiol. 2024;413:1–11. 10.1016/j.ijfoodmicro.2024.110587 38301541

[bib12] Carrascosa C, Raheem D, Ramos F et al. Microbial biofilms in the food industry—a comprehensive review. Int J Environ Res Public Health. 2021;18:2014. 10.3390/ijerph18042014 33669645 PMC7922197

[bib13] Chang SS, Han A, Reyes-De-Corcuera J et al. Evaluation of steam pasteurization in controlling *Salmonella* serotype Enteritidis on raw almond surfaces. Lett Appl Microbiol. 2010;50:393–8. 10.1111/j.1472-765X.2010.02809.x 20184671

[bib14] Cristobal-Cueto P, García-Quintanilla A, Esteban J et al. Phages in food industry biocontrol and bioremediation. Antibiotics. 2021;10:786. 10.3390/antibiotics10070786 34203362 PMC8300737

[bib15] Duc HM, Son HM, Yi HPS et al. Isolation, characterization and application of a polyvalent phage capable of controlling *Salmonella* and *Escherichia coli* O157:H7 in different food matrices. Food Res Int. 2020;131:108977. 10.1016/j.foodres.2020.108977 32247506

[bib16] El-Dougdoug NK, Cucic S, Abdelhamid AG et al. Control of *Salmonella* Newport on cherry tomato using a cocktail of lytic bacteriophages. Int J Food Microbiol. 2019;293:60–71. 10.1016/j.ijfoodmicro.2019.01.003 30641253

[bib18] European Food Safety Authority (EFSA). Food Zoonotic Diseases. 2023. https://www.efsa.europa.eu/en/topics/topic/foodborne-zoonotic-diseases (4th April 2025, date last accessed).

[bib17] European Food Safety Authority (EFSA). Panel on Biological Hazards (BIOHAZ). Evaluation of the safety and efficacy of Listex^TM^ P100 for reduction of pathogens on different ready-to-eat (RTE) food products. EFSA J. 2016;14:e04565. 10.2903/j.efsa.2016.4565

[bib19] European Food Safety Authority (EFSA). *Public health aspects of* Vibrio *spp. related to the consumption of seafood in the EU*. 2024. https://www.efsa.europa.eu/en/efsajournal/pub/8896 (4th April 2025, date last accessed). 10.2903/j.efsa.2024.8896 PMC11263920 39045511

[bib20] Ferguson S, Roberts C, Handy E et al. Lytic bacteriophages reduce *Escherichia coli* O157: H7 on fresh cut lettuce introduced through cross-contamination. Bacteriophage. 2013;3:e24323. 10.4161/bact.24323 23819106 PMC3694057

[bib21] Fernebro J. Fighting bacterial infections—future treatment options. Drug Resist Updat. 2011;14:125–39. 10.1016/j.drup.2011.02.001 21367651

[bib22] Folsom JP, Frank JF. Chlorine resistance of *Listeria monocytogenes* biofilms and relationship to subtype, cell density, and planktonic cell chlorine resistance. J Food Prot. 2006;69:1292–6. 10.4315/0362-028X-69.6.1292 16786848

[bib23] Food and Drug Administration (FDA) Retail Food Protection: Employee Health and Personal Hygiene Handbook. 2022. https://www.fda.gov/food/retail-food-industryregulatory-assistance-training/retail-food-protection-employee-health-and personal-hygiene-handbook (4th April 2025, date last accessed).

[bib25] Garvey M. Bacteriophages and food production: biocontrol and bio-preservation options for food safety 2022. Antibiotics. 2022;11:1324. 10.3390/antibiotics1110132 36289982 PMC9598955

[bib26] Gizaw Z. Public health risks related to food safety issues in the food market: a systematic literature review. Environ Health Prev Med. 2019;24:68. https://doi.10.1186/s12199-019-0825-5 31785611 10.1186/s12199-019-0825-5 PMC6885314

[bib27] González-Gómez P, González-Torres B, Guerrero-Medina PJ et al. Efficacy of novel bacteriophages against *Escherichia coli* biofilms on stainless steel. Antibiotics. 2021;10:1–13. 10.3390/antibiotics10101150 PMC8532843 34680731

[bib28] Gray JA, Chandry PS, Kaur M et al. Novel biocontrol methods for *Listeria monocytogenes* biofilms in food production facilities. Front Microbiol. 2018;9:605. https://doi.10.3389/fmicb.2018.00605 29666613 10.3389/fmicb.2018.00605 PMC5891606

[bib29] Guenther S, Herzig O, Fieseler L et al. Biocontrol of *Salmonella* Typhimurium in RTE foods with the virulent bacteriophage FO1-E2. Int J Food Microbiol. 2012;154:66–72. 10.1016/j.ijfoodmicro.2011.12.023 22244192

[bib30] Guenther S, Loessner MJ. Bacteriophage biocontrol of *Listeria monocytogenes* on soft ripened white mold and red-smear cheeses. Bacteriophage. 2011;1:94–100. 10.4161/bact.1.2.15662 22334865 PMC3278646

[bib31] Gutiérrez D, Rodríguez-Rubio L, Fernández L et al. Applicability of commercial phage-based products against *Listeria monocytogenes* for improvement of food safety in Spanish dry-cured ham and food contact surfaces. Food Control. 2017;73:1474–82. 10.1016/j.foodcont.2016.11.007

[bib32] Hungaro HM, Mendonça RCS, Gouvêa DM et al. Use of bacteriophages to reduce *Salmonella* in chicken skin in comparison with chemical agents. Food Res Int. 2013;52:75–81. 10.1016/j.foodres.2013.02.032

[bib33] Iacumin L, Manzano M, Comi G. Phage inactivation of *Listeria monocytogenes* on San Daniele dry-cured ham and elimination of biofilms from equipment and working environments. Microorganisms. 2016;4:4. 10.3390/microorganisms4010004 27681898 PMC5029509

[bib34] Islam MS, Zhou Y, Liang L et al. Application of a phage cocktail for control of *Salmonella* in foods and reducing biofilms. Viruses. 2019;11:841. 10.3390/v11090841 31510005 PMC6784009

[bib35] Jaroni D, Litt PK, Bule P et al. Effectiveness of bacteriophages against biofilm-forming Shiga-toxigenic *Escherichia coli* in vitro and on food-contact surfaces. Foods. 2023;12:1–14. 10.3390/foods12142787 PMC10378794 37509879

[bib36] Jun JW, Kim HJ, Yun SK et al. Eating oysters without risk of vibriosis: application of a bacteriophage against *Vibrio parahaemolyticus* in oysters. Int J Food Microbiol. 2014;188:31–35. 10.1016/j.ijfoodmicro.2014.07.007 25086350

[bib37] Kasman LM, Porter LD. Bacteriophages. StatPearls Publishing, 2024. https://www.ncbi.nlm.nih.gov/books/NBK493185/ (12 December 2024, date last accessed). 29630237

[bib38] Kawacka I, Olejnik-Schmidt A, Schmidt M et al. Effectiveness of phage-based inhibition of *Listeria monocytogenes* in food products and food processing environments. Microorganisms. 2020;8:1764. 10.3390/microorganisms8111764 33182551 PMC7697088

[bib39] Khan JA, Husain FM, Gezahegne G et al. Biofilm in antibiotic resistance and pathogenesis in relation to foodborne infection and control strategies. In: Das S, Kungwani NA (eds), Understanding Microbial Biofilms, Chapter 19. Academic Press, 2023, 315–34. 10.1016/B978-0-323-99977-9.00017-X

[bib40] Kim M, Weigand MR, Oh S et al. Widely used Benzalkonium chloride disinfectants can promote antibiotic resistance. Appl Environ Microb. 2018;84:e01201–18. 10.1128/AEM.01201-18 PMC6102991 29959242

[bib41] Kim SH, Park SH, Kim SS et al. Inactivation of *Staphylococcus aureus* biofilms on food contact surfaces by superheated steam treatment. J Food Prot. 2019;82:1496–500. 10.4315/0362-028X.JFP-18-572 31411506

[bib42] Kim WJ, Kim SH, Kang DH. Thermal and non-thermal treatment effects on *Staphylococcus aureus* biofilms formed at different temperatures and maturation periods. Food Res Int. 2020;137:1–8. 10.1016/j.foodres.2020.109432/33233114

[bib43] Kokab A, Sheikh AA, Rabbani M et al. Physiological profiling of lytic bacteriophages and their efficiency against *Salmonella* Enteritidis on chicken breast cuts. PJZ. 2022;56:1–10. 10.17582/journal.pjz/20220607110620

[bib44] Kutateladze M, Adamia R. Bacteriophages as potential new therapeutics to replace or supplement antibiotics. Trends Biotechnol. 2010;28:591–5. 10.1016/j.tibtech.2010.08.001 20810181

[bib45] Lambuk F, Mazlan N, Lesen D et al. *Staphylococcus aureus* lytic bacteriophage: Isolation and application evaluation. J Consum Prot Food Saf. 2024;19:235–43. 10.1007/s00003-024-01479-8

[bib46] Liu H, Niu YD, Meng R et al. Control of *Escherichia coli* O157 on beef at 37, 22 and 4°C by T5-, T1-, T4-and O1-like bacteriophages. Food Microbiol. 2015;51:69–73. 10.1016/j.fm.2015.05.001 26187829

[bib47] Liu X, Yao H, Zhao X et al. Biofilm formation and control of foodborne pathogenic bacteria. Molecules. 2023;28:2432. 10.3390/molecules28062432 36985403 PMC10058477

[bib48] Lu YT, Ma Y, Wong CW et al. Characterization and application of bacteriophages for the biocontrol of Shiga-toxin producing *Escherichia coli* in romaine lettuce. Food Control. 2022;140:1–16. 10.1016/j.foodcont.2022.109109

[bib49] Magnone JP, Marek PJ, Sulakvelidze A et al. Additive approach for inactivation of *Escherichia coli* O157:H7, *Salmonella*, and *Shigella* spp. on contaminated fresh fruits and vegetables using bacteriophage cocktail and produce wash. J Food Prot. 2013;76:1336–41. 10.4315/0362-028X.JFP-12-517 23905788

[bib50] Meaden S, Koskella B. Exploring the risks of phage application in the environment. Front Microbiol. 2013;4:358. 10.3389/fmicb.2013.00358 24348468 PMC3843224

[bib51] Montañez-Izquierdo VY, Salas-Vázquez DI, Rodríguez-Jerez JJ. Use of epifluorescence microscopy to assess the effectiveness of phage P100 in controlling *Listeria monocytogenes* biofilms on stainless steel surfaces. Food Control. 2012;23:470–7. 10.1016/j.foodcont.2011.08.016

[bib53] Moye ZD, Woolston J, Sulakvelidze A. Bacteriophage applications for food production and processing. Viruses. 2018;10:205. 10.3390/v10040205 29671810 PMC5923499

[bib54] Noah D. Incidence and Impact of Foodborne Illness MSD Veterinary Manual 2022/12. 2022. https://www.msdvetmanual.com/public-health/food-safety/incidence-and-impact-of-foodborne-illness (12 December, date last accessed).

[bib56] O'Sullivan JN. Demographic delusions: world population growth is exceeding most projections and jeopardising scenarios for sustainable futures. WORLD. 2023;4: 545–68. 10.3390/world4030034

[bib55] Oliveira M, Viñas I, Colàs P et al. Effectiveness of a bacteriophage in reducing *Listeria monocytogenes* on fresh-cut fruits and fruit juices. Food Microbiol. 2014;38:137–42. 10.1016/j.fm.2013.08.018 24290636

[bib57] Page MJ, McKenzie JE, Bossuyt PM et al. The PRISMA 2020 statement: an updated guideline for reporting systematic reviews. J Clin Epidemiol. 2021;134:178–89. 10.1016/j.jclinepi.2021.03.001 33789819

[bib58] Pang X, Hyun-Gyun Y. Effects of the colonization sequence of *Listeria monocytogenes* and *Pseudomonas fluorescens* on survival of biofilm cells under food-related stresses and transfer to salmon. Food Microbiol. 2019;82:142–50. 10.1016/j.fm.2019.02.002 31027768

[bib59] Park HW, Yoon WB. A quantitative microbiological exposure assessment model for *Bacillus cereus* in pasteurized rice cakes using computational fluid dynamics and monte carlo simulation. Food Res Int. 2019;125:108562. 10.1016/j.foodres.2019.108562 31554100

[bib60] Pinto G, Minnich SA, Hovde CJ et al.‘The interactions of bacteriophage Ace and Shiga toxin-producing *Escherichia coli* during biocontrol. FEMS Microbiol Ecol. 2021;97:1–15. 10.1093/femsec/fiab105 PMC8492476 34329454

[bib61] Randall L, Cooles S, Coldham N et al. Commonly used farm disinfectants can select for mutant *Salmonella enterica* serovar Typhimurium with decreased susceptibility to biocides and antibiotics without compromising virulence. J Antimicrob Chemother. 2007;60:1273–80. 10.1093/jac/dkm359 17897935

[bib62] Sadekuzzaman M, Mizan MFR, Yang S et al. Application of bacteriophages for the inactivation of *Salmonella* spp. in biofilms. Food Sci Technol Int. 2018;24:424–33. 10.1177/1082013218763424 29546997

[bib64] Sadekuzzaman M, Yang S, Mizan MFR et al. Effectiveness of a phage cocktail as a biocontrol agent against *L. monocytogenes* biofilms. Food Control. 2017a;78:256–63. 10.1016/j.foodcont.2016.10.056

[bib63] Sadekuzzaman M, Yang S, Mizan MFR et al. Reduction of *Escherichia coli* O157: H7 in biofilms using bacteriophage BPECO 19. J Food Sci. 2017b;82:1433–42. 10.1111/1750-3841.13729 28542913

[bib65] Salmond GPC, Fineran PC. A century of the phage: past, present and future. Nat Rev Micro. 2015;13:777–86. 10.1038/nrmicro3564 26548913

[bib66] Shi X, Zhu X. Biofilm formation and food safety in food industries. Trends Food Sci Technol. 2009;20:407–13. 10.1016/j.tifs.2009.01.054

[bib67] Silva EN, Figueiredo AC, Miranda FA et al. Control of *Listeria monocytogenes* growth in soft cheeses by bacteriophage P100. Braz J Microbiol. 2014;45:11–16. 10.1590/S1517-83822014000100003 24948908 PMC4059284

[bib68] Siringan P, Connerton PL, Payne RJ. Bacteriophage-mediated dispersal of *Campylobacter jejuni* biofilms.’, Appl Environ Microb. 2011;77:3320–6. 10.1128/AEM.02704-10 PMC3126433 21441325

[bib69] Sofy AR, Abd El Haliem NF, Refaey EE et al. Polyvalent phage CoNShP-3 as a natural antimicrobial agent showing lytic and antibiofilm activities against antibiotic-resistant coagulase-negative staphylococci strains. Foods. 2020;9:1–24. 10.3390/foods9050673 PMC7278617 32456227

[bib70] Song J, Ruan H, Chen L et al. Potential of bacteriophages as disinfectants to control of *Staphylococcus aureus* biofilms. BMC Microbiol. 2021;21:57. 10.1186/s12866-021-02117 33607940 PMC7896381

[bib71] Soni KA, Nannapaneni R, Hagens S. Reduction of *Listeria monocytogenes* on the surface of fresh channel catfish fillets by bacteriophage Listex P100. Foodborne Pathog Dis. 2010;7:427–34. 10.1089/fpd.2009.0432 19958102

[bib72] Soni KA, Nannapaneni R. Bacteriophage significantly reduces *Listeria monocytogenes* on raw salmon fillet tissue. J Food Prot. 2010b;73:32–38. 10.4315/0362-028X-73.1.32 20051201

[bib73] Soni KA, Nannapaneni R. Removal of *Listeria monocytogenes* biofilms with bacteriophage P100. J Food Prot. 2010a;73:1519–24. 10.4315/0362-028X-73.8.1519 20819365

[bib74] Spricigo DA, Bardina C, Cortés P et al. Use of a bacteriophage cocktail to control Salmonella in food and the food industry. Int J Food Microbiol. 2013;165:169–74. 10.1016/j.ijfoodmicro.2013.05.009 23735218

[bib75] Sukumaran AT, Nannapaneni R, Kiess A et al. Reduction of *Salmonella* on chicken meat and chicken skin by combined or sequential application of lytic bacteriophage with chemical antimicrobials. Int J Food Microbiol. 2015;207:8–15. 10.1016/j.ijfoodmicro.2015.04.025 25950852

[bib76] Tavares J, Martins A, Fidalgo LG et al. Fresh fish degradation and advances in preservation using physical emerging technologies. Foods. 2021;10:780. 10.3390/foods10040780 33916441 PMC8066737

[bib77] Thung TY, Krishanthi Jayarukshi Kumari Premarathne JM, San Chang W et al. Use of a lytic bacteriophage to control *Salmonella* Enteritidis in retail food. Microorganisms. 2017;4:222–5. 10.3390/microorganisms4010004

[bib78] Thung TY, Lee E, Mahyudin NA et al. Partial characterization and in vitro evaluation of a lytic bacteriophage for biocontrol of *Campylobacter jejuni* in mutton and chicken meat. J Food Saf. 2020;40:e12770. 10.1111/jfs.12770

[bib79] U.S. Food & Drug Administration (FDA). GRAS Notice No. 198. 2024a. https://www.cfsanappsexternal.fda.gov/scripts/fdcc/index.cfm?set=GRASNotices&id=198 (4th April 2025, date last accessed).

[bib80] U.S. Food & Drug Administration (FDA). GRAS Notice No. 218. 2024b. https://www.cfsanappsexternal.fda.gov/scripts/fdcc/index.cfm?set=GRASNotices&id=218 (4th April 2025, date last accessed).

[bib81] U.S. Food & Drug Administration (FDA). GRAS Notice No. 528. 2024c. https://www.cfsanappsexternal.fda.gov/scripts/fdcc/index.cfm?set=GRASNotices&id=528 (4th April 2025, date last accessed).

[bib83] Wang C, Hang H, Zhou S et al. Bacteriophage biocontrol of Shiga toxigenic *Escherichia coli* (STEC) O145 biofilms on stainless steel reduces the contamination of beef. Food Microbiol. 2020;92:1–9. 10.1016/j.fm.2020.103572 32950157

[bib84] Wang H, Zhang X, Zhang Q et al. Comparison of microbial transfer rates from *Salmonella spp*. biofilm growth on stainless steel to selected processed and raw meat. Food Control. 2015;50:574–80. 10.1016/j.foodcont.2014.09.049

[bib86] World Health Organization (WHO). Antimicrobial Resistance. 2024. https://www.who.int/news-room/fact-sheets/detail/antimicrobial-resistance (4th April 2025, date last accessed).

[bib85] World Health Organization (WHO). Food Safety. 2022. https://www.who.int/news-room/fact-sheets/detail/food-safety (4th April 2025, date last accessed).

[bib87] Yang S., Sadekuzzaman M, Ha SD. Reduction of *Listeria monocytogenes* on chicken breasts by combined treatment with UV-C light and bacteriophage ListShield. LWT. 2017;86:193–200. 10.1016/j.lwt.2017.07.060

[bib88] Yassoralipour A, Wong JX, Chow WH et al. Simulated transmission and decontamination of *Listeria monocytogenes* biofilms from plastic cutting boards. Food Control. 2023;149:109678. 10.1016/j.foodcont.2023.109678

[bib89] Yeh Y, Purushothaman P, Gupta N et al. Bacteriophage application on red meats and poultry: Effects on *Salmonella* population in final ground products. Meat Sci. 2017;127:30–34. 10.1016/j.meatsci.2017.01.001 28110127

[bib90] You H, Lee JH, Oh M et al. Tackling *Vibrio parahaemolyticus* in ready-to-eat raw fish flesh slices using lytic phage VPT02 isolated from market oyster. Food Res Int. 2021;150:1–10. 10.1016/j.foodres.2021.110779 34865794

[bib91] Zampara A, Sørensen MCH, Elsser-Gravesen A et al. Significance of phage-host interactions for biocontrol of *Campylobacter jejuni* in food. Food Control. 2017;73:1169–75. 10.1016/j.foodcont.2016.10.033

[bib92] Zhang H, Yang Z, Zhou Y et al. Application of a phage in decontaminating *Vibrio parahaemolyticus* in oysters. Int J Food Microbiol. 2018;275:24–31. 10.1016/j.ijfoodmicro.2018.03.027 29621738

