# Supplementary material for: Use of bacteriophages for biocontrol of pathogens in food and food-contact surfaces: a systematic review of the literature
Source: Sustain Microbiol. 2025 Apr 29;2(1):qvaf005. doi: 10.1093/sumbio/qvaf005 (PMC13375297; doi:10.1093/sumbio/qvaf005)
Supplement: Supplementary file 1 [file qvaf005_Supplemental_File.docx]

## **List of final 49 scientific studies selected for this systematic review of the literature.**

1. Aguilera M, Tobar-Calfucoy E, Rojas-Martínez V, Norambuena R, Serrano MJ, Cifuentes, O, Zamudio MS, San Martín D, Lara P, Sabag A and Zabner M. Development and characterization of a bacteriophage cocktail with high lytic efficacy against field-isolated *Salmonella enterica*.  *Poultry Sci* 2023; 102:1-14. <https://doi.org/10.1016/j.psj.2023.103125>.
2. Allué-Guardia A, Martínez-Castillo A and Muniesa M. Persistence of infectious Shiga toxin-encoding bacteriophages after disinfection treatments.  *App Environ Microbiol* 2014;  80: 2142-2149 <https://doi.org/10.1128/AEM.04006-13>.
3. Ashrafudoulla M, Kim, HJ, Her E, Shaila S, Park SH and Ha SD. Characterization of *Salmonella* Thompson-specific bacteriophages and their preventive application against *Salmonella* Thompson biofilm on eggshell as a promising antimicrobial agent in the food industry.  *Food Control* 2023; 154:1-12. <https://doi.org/10.1016/j.foodcont.2023.110008>
4. Bao H, Zhang P, Zhang H, Zhou Y, Zhang L, Wang R. Bio-Control of Salmonella Enteritidis in Foods Using Bacteriophages. *Viruses* 2015; 7:4836-53. <https://doi.org/10.3390/v7082847>
5. Bigot B, Lee WJ, McIntyre L, Wilson T, Hudson JA, Billington C, Heinemann JA. Control of Listeria monocytogenes growth in a ready-to-eat poultry product using a bacteriophage. *Food Microbiol* 2011; 28:1448-52. <https://doi.org/10.1016/j.fm.2011.07.001>.
6. Byun KH, Han SH, Choi MW, Kim BH and Ha SD. Efficacy of disinfectant and bacteriophage mixture against planktonic and biofilm state of *Listeria monocytogenes* to control in the food industry.  *Int J Food Microbiol* 2024; 413:1-11. <https://doi.org/10.1016/j.ijfoodmicro.2024.110587>.
7. Duc HM, Son HM, Yi HPS, Sato J, Ngan PH, Masuda Y, Honjoh KI, Miyamoto T. Isolation, characterization and application of a polyvalent phage capable of controlling Salmonella and Escherichia coli O157:H7 in different food matrices. *Food Res Int* 2020; 131:108977. <https://doi.org/10.1016/j.foodres.2020.108977>.
8. El-Dougdoug NK, Cucic S, Abdelhamid AG, Brovko L, Kropinski AM, Griffiths MW. and Anany, H. Control of *Salmonella* Newport on cherry tomato using a cocktail of lytic bacteriophages. *Int J Food Microbiol* 2019; 293:60-71. <https://doi.org/10.1016/j.ijfoodmicro.2019.01.003>.
9. Ferguson S, Roberts C, Handy E, Sharma M. Lytic bacteriophages reduce Escherichia coli O157: H7 on fresh cut lettuce introduced through cross-contamination. *Bacteriophage* 2013; 3:e24323. <https://doi.org/10.4161/bact.24323>.
10. Ganegama Arachchi GJ, Cridge AG, Dias-Wanigasekera BM, Cruz CD, McIntyre L, Liu R, Flint SH, Mutukumira AN. Effectiveness of phages in the decontamination of Listeria monocytogenes adhered to clean stainless steel, stainless steel coated with fish protein, and as a biofilm. *J Ind Microbiol Biotechnol* 2013; 40:1105-16. <https://doi.org/10.1007/s10295-013-1313-3>.
11. González-Gómez P, González-Torres B, Guerrero-Medina PJ, López-Cuevas O, Chaidez C, Avila-Novoa MG and Gutiérrez-Lomelí M. Efficacy of novel bacteriophages against *Escherichia coli* biofilms on stainless steel.  *Antibiotics* 2021;  10:1-13. <https://doi.org/10.3390/antibiotics10101150>.
12. Guenther S, Herzig O, Fieseler L, Klumpp J, Loessner MJ. Biocontrol of Salmonella Typhimurium in RTE foods with the virulent bacteriophage FO1-E2. *Int J Food Microbiol* 2012; 154:66-72. <https://doi.org/10.1016/j.ijfoodmicro.2011.12.023>.
13. Guenther S and Loessner MJ. Bacteriophage biocontrol of Listeria monocytogenes on soft ripened white mold and red-smear cheeses. *Bacteriophage* 2011. 1:94-100. <https://doi.org/10.4161/bact.1.2.15662>.
14. Gutiérrez D, Rodríguez-Rubio L, Fernández L, Martínez B, Rodríguez A and García P. Applicability of commercial phage-based products against *Listeria monocytogenes* for improvement of food safety in Spanish dry-cured ham and food contact surfaces. *Food Control* 2017;  73:1474-1482. <https://doi.org/10.1016/j.foodcont.2016.11.007>.
15. Hungaro HM, Mendonça RCS, Gouvêa DM, Vanetti MCD and de Oliveira Pinto CL. Use of bacteriophages to reduce *Salmonella* in chicken skin in comparison with chemical agents. *Food Res Int* 2013; 52:75–81. <https://doi.org/10.1016/j.foodres.2013.02.032>.
16. Iacumin L, Manzano M and Comi G. Phage Inactivation of Listeria monocytogenes on San Daniele Dry-Cured Ham and Elimination of Biofilms from Equipment and Working Environments. *Microorganisms* 2016; 4:4. [https://doi.org/10.3390/microorganisms4010004](https://doi.org/10.3390/microorganisms4010004%20).
17. Islam MS, Zhou Y, Liang L, Nime I, Liu K, Yan T, Wang X and Li J. Application of a Phage Cocktail for Control of *Salmonella* in Foods and Reducing Biofilms. *Viruses* 2019. 11(9):841. <https://doi.org/10.3390/v11090841>.
18. Jaroni D, Litt PK, Bule P and Rumbaugh K. Effectiveness of bacteriophages against biofilm-forming Shiga-toxigenic *Escherichia coli* in vitro and on food-contact surfaces.  *Foods* 2023;  12:1-14. <https://doi.org/10.3390/foods12142787>.
19. Jun JW, Kim HJ, Yun SK, Chai JY and Park SC. Eating oysters without risk of vibriosis: application of a bacteriophage against Vibrio parahaemolyticus in oysters. *Int J Food Microbiol* 2014; 188:31-5. <https://doi.org/10.1016/j.ijfoodmicro.2014.07.007>.
20. Kokab A, Sheikh AA, Rabbani M, Shehzad W, Riaz MI, Raza S. and Durrani RH. Physiological profiling of lytic bacteriophages and their efficiency against Salmonella Enteritidis on chicken breast cuts. *Pakistan J Zool* 2022; 56:1-10. <https://dx.doi.org/10.17582/journal.pjz/20220607110620>.
21. Lambuk F, Mazlan N, Lesen D, Nillian E and Thung TY. *Staphylococcus aureus* lytic bacteriophage: isolation and application evaluation.  *J Cons Prot Food S* 2024; 19:1-9. <https://doi.org/10.1007/s00003-024-01479-8>.
22. Liu H, Niu YD, Meng R, Wang J, Li J, Johnson RP, McAllister TA and Stanford K. Control of Escherichia coli O157 on beef at 37, 22 and 4 °C by T5-, T1-, T4-and O1-like bacteriophages. *Food Microbiol* 2015; 51:69-73. <https://doi.org/10.1016/j.fm.2015.05.001>.
23. Lu YT, Ma Y, Wong CW and Wang S. Characterization and application of bacteriophages for the biocontrol of Shiga-toxin producing *Escherichia coli* in romaine lettuce. *Food Control* 2022; 140:1-16. <https://doi.org/10.1016/j.foodcont.2022.109109>.
24. Magnone JP, Marek PJ, Sulakvelidze A and Senecal AG. Additive approach for inactivation of Escherichia coli O157:H7, Salmonella, and Shigella spp. on contaminated fresh fruits and vegetables using bacteriophage cocktail and produce wash. *J Food Prot* 2013. 76:1336-41. <https://doi.org/10.4315/0362-028X.JFP-12-517>.
25. Montañez-Izquierdo VY, Salas-Vázquez DI and Rodríguez-Jerez JJ. Use of epifluorescence microscopy to assess the effectiveness of phage P100 in controlling *Listeria monocytogenes* biofilms on stainless steel surfaces. *Food Control* 2012; 23:470–477. [https://doi.org/10.1016/j.foodcont.2011.08.016](https://doi.org/10.1016/j.foodcont.2011.08.016%20).
26. Montso PK, Mlambo V and Ateba, CN. Efficacy of novel phages for control of multi-drug resistant *Escherichia coli* O177 on artificially contaminated beef and their potential to disrupt biofilm formation. *Food Microbiol* 2021; 94:1-10. <https://doi.org/10.1016/j.fm.2020.103647>.
27. Oliveira M, Viñas I, Colàs P, Anguera M, Usall J, Abadias M. Effectiveness of a bacteriophage in reducing Listeria monocytogenes on fresh-cut fruits and fruit juices. *Food Microbiol* 2014. 38:137-42. <https://doi.org/10.1016/j.fm.2013.08.018>.
28. Pinto G, Minnich SA, Hovde CJ, Oliveira H, Smidt H, Almeida C and Azeredo J. ‘The interactions of bacteriophage Ace and Shiga toxin-producing *Escherichia coli* during biocontrol. *FEMS Microbiol Ecol* 2021; 97:1-15. <https://doi.org/10.1093/femsec/fiab105>.
29. Sadekuzzaman M, Mizan MFR, Yang S, Kim HS and Ha SD. Application of bacteriophages for the inactivation of *Salmonella* spp. in biofilms. *Food Sci Tech Int*  2018; 24:424-433. <https://doi.org/10.1177/1082013218763424>.
30. Sadekuzzaman M, Yang S, Mizan MFR, Kim HS and Ha SD. Effectiveness of a phage cocktail as a biocontrol agent against *L. monocytogenes* biofilms. *Food Control* 2017a; 78:256-263. <https://doi.org/10.1016/j.foodcont.2016.10.056>.
31. Sadekuzzaman M, Yang S, Mizan MFR and Ha SD. Reduction of *Escherichia coli* O157: H7 in biofilms using bacteriophage BPECO 19. *J Food Sci* 2017b; 82:1433-1442. <https://doi.org/10.1111/1750-3841.13729>.
32. Silva EN, Figueiredo AC, Miranda FA and de Castro Almeida RC. Control of Listeria monocytogenes growth in soft cheeses by bacteriophage P100. *Braz J Microbiol* 2014; 45:11-6. <https://doi.org/10.1590/s1517-83822014000100003>.
33. Siringan P, Connerton PL and Payne RJ. Bacteriophage-mediated dispersal of *Campylobacter jejuni* biofilms’, *Appl Environ Microbiol* 2011; 77:3320-3326. <https://doi.org/10.1128/AEM.02704-10>.
34. Sofy AR, Abd El Haliem NF, Refaey EE and Hmed AA. Polyvalent phage CoNShP-3 as a natural antimicrobial agent showing lytic and antibiofilm activities against antibiotic-resistant coagulase-negative staphylococci strains. *Foods* 2020; 9:1-24. <https://doi.org/10.3390/foods9050673>.
35. Soni KA and Nannapaneni R. 2010a. Removal of Listeria monocytogenes biofilms with bacteriophage P100. J Food Prot. 2010a; 73:1519-24. [https://doi.org/10.4315/0362-028x-73.8.1519.](https://doi.org/10.4315/0362-028x-73.8.1519.%20)
36. Soni KA and Nannapaneni R. Bacteriophage Significantly Reduces *Listeria monocytogenes* on Raw Salmon Fillet Tissue. *J Food Prot* 2010b; 73:32–38. <https://doi.org/10.4315/0362-028X-73.1.32>.
37. Soni KA, Nannapaneni R and Hagens S. Reduction of Listeria monocytogenes on the surface of fresh channel catfish fillets by bacteriophage Listex P100. *Foodborne Pathog Dis* 2010; 7:427-34. <https://doi.org/10.1089/fpd.2009.0432>.
38. Song J, Ruan H, Chen L, Jin Y, Zheng J, Wu R and Sun D. Potential of bacteriophages as disinfectants to control of Staphylococcus aureus biofilms. *BMC Microbiol* 2021; 21:57. <https://doi.org/10.1186/s12866-021-02117>.
39. Spricigo DA, Bardina C, Cortés P and Llagostera M. Use of a bacteriophage cocktail to control Salmonella in food and the food industry. *Int J Food Microbiol* 2013; 165:169-74. <https://doi.org/1016/j.ijfoodmicro.2013.05.009>.
40. Sukumaran AT, Nannapaneni R, Kiess A and Sharma CS. Reduction of Salmonella on chicken meat and chicken skin by combined or sequential application of lytic bacteriophage with chemical antimicrobials. *Int J Food Microbiol* 2015; 207:8-15. <https://doi.org/10.1016/j.ijfoodmicro.2015.04.025>.
41. Thung TY, Lee E, Mahyudin NA, Radzi CWJWM, Mazlan N, Tan CW and Radu S. Partial characterization and in vitro evaluation of a lytic bacteriophage for biocontrol of *Campylobacter jejuni* in mutton and chicken meat. *J. Food Saf* 2019; 40:e12770. <https://doi.org/10.1111/jfs.12770>.
42. Thung TY , Krishanthi Jayarukshi Kumari Premarathne JM , San Chang W, Loo YY , Chin, YZ , Kuan CH, Tan CW, Basri DF, Jasimah Wan Mohamed Radzi CW and Radu S. Use of a lytic bacteriophage to control Salmonella Enteritidis in retail food. LWT - *Food Sci Tech* 2017; 78:222–225. <https://doi.org/10.3390/microorganisms4010004>.
43. Vongkamjan K, Roof S, Stasiewicz MJ and Wiedmann M. Persistent *Listeria monocytogenes* subtypes isolated from a smoked fish processing facility included both phage susceptible and resistant isolates. *Food Microbiol* 2013;  35:38-48. <https://doi.org/10.1016/j.fm.2013.02.012>.
44. Wang C, Hang H, Zhou S, Niu YD, Du H, Stanford K and McAllister TA. Bacteriophage biocontrol of Shiga toxigenic *Escherichia coli* (STEC) O145 biofilms on stainless steel reduces the contamination of beef*. Food Microbiol* 2020; 92:1-9. <https://doi.org/10.1016/j.fm.2020.103572>.
45. Yang S. Sadekuzzaman M and Ha SD. Reduction of *Listeria monocytogenes* on chicken breasts by combined treatment with UV-C light and bacteriophage ListShield. LWT - Food Sci Tech 2017; 86:193–200. <https://doi.org/10.1016/j.lwt.2017.07.060>.
46. Yeh Y, Purushothaman P, Gupta N, Ragnone M, Verma SC and de Mello AS. Bacteriophage application on red meats and poultry: Effects on Salmonella population in final ground products. *Meat Sci* 2017; 127:30-34. <https://doi.org/10.1016/j.meatsci.2017.01.001>.
47. You H., Lee JH, Oh M, Hong SY, Kim D, Noh J, Kim M and Kim BS. Tackling *Vibrio parahaemolyticus* in ready-to-eat raw fish flesh slices using lytic phage VPT02 isolated from market oyster. *Food Res Int* 2021; 150:1-10. <https://doi.org/10.1016/j.foodres.2021.110779>.
48. Zampara A, Sørensen MCH, Elsser-Gravesen A and Brøndsted L. Significance of phage-host interactions for biocontrol of *Campylobacter jejuni* in food. *Food Control* 2017; 73:1169–75. <https://doi.org/10.1016/j.foodcont.2016.10.033>.
49. Zhang H, Yang Z, Zhou Y, Bao H, Wang R, Li T, Pang M, Sun L, Zhou X. Application of a phage in decontaminating Vibrio parahaemolyticus in oysters. *Int J Food Microbiol* 2018; 275:24-31. <https://doi.org/10.1016/j.ijfoodmicro.2018.03.027>
